# Maternal and Newborn Health in Karnataka State, India: The Community Level Interventions for Pre-Eclampsia (CLIP) Trial’s Baseline Study Results

**DOI:** 10.1371/journal.pone.0166623

**Published:** 2017-01-20

**Authors:** Mrutynjaya B. Bellad, Marianne Vidler, Narayan V. Honnungar, Ashalata Mallapur, Umesh Ramadurg, Umesh Charanthimath, Geetanjali Katageri, Shashidhar Bannale, Avinash Kavi, Chandrashekhar Karadiguddi, Sumedha Sharma, Tang Lee, Jing Li, Beth Payne, Laura Magee, Peter von Dadelszen, Richard Derman, Shivaprasad S. Goudar, CLIP Working Group

**Affiliations:** 1 Women’s and Children’s Health Research Unit, KLE’s Jawaharlal Nehru Medical College, Belgaum, Karnataka, India; 2 Department of Obstetrics and Gynaecology, University of British Columbia, Vancouver, British Columbia, Canada; 3 S Nijalingappa Medical College, Balgalkot, Karnataka, India; 4 Molecular and Clinical Sciences Research Institute, St George’s, University of London and Department of Obstetrics and Gynaecology, St George’s University Hospitals NHS Foundation Trust, London, United Kingdom; 5 Department Obstetrics and Gynaecology and Global Health Research Thomas Jefferson University, Philadelphia, Pennsylvania, United States of America; Yale School of Public Health, UNITED STATES

## Abstract

Existing vital health statistics registries in India have been unable to provide reliable estimates of maternal and newborn mortality and morbidity, and region-specific health estimates are essential to the planning and monitoring of health interventions. This study was designed to assess baseline rates as the precursor to a community-based cluster randomized control trial (cRCT)–Community Level Interventions for Pre-eclampsia (CLIP) Trial (NCT01911494; CTRI/2014/01/004352). The objective was to describe baseline demographics and health outcomes prior to initiation of the CLIP trial and to improve knowledge of population-level health, in particular of maternal and neonatal outcomes related to hypertensive disorders of pregnancy, in northern districts the state of Karnataka, India. The prospective population-based survey was conducted in eight clusters in Belgaum and Bagalkot districts in Karnataka State from 2013–2014. Data collection was undertaken by adapting the Maternal and Newborn Health registry platform, developed by the Global Network for Women’s and Child Health Studies. Descriptive statistics were completed using SAS and R. During the period of 2013–2014, prospective data was collected on 5,469 pregnant women with an average age of 23.2 (+/-3.3) years. Delivery outcomes were collected from 5,448 completed pregnancies. A majority of the women reported institutional deliveries (96.0%), largely attended by skilled birth attendants. The maternal mortality ratio of 103 (per 100,000 livebirths) was observed during this study, neonatal mortality ratio was 25 per 1,000 livebirths, and perinatal mortality ratio was 50 per 1,000 livebirths. Despite a high number of institutional deliveries, rates of stillbirth were 2.86%. Early enrollment and close follow-up and monitoring procedures established by the Maternal and Newborn Health registry allowed for negligible lost to follow-up. This population-level study provides regional rates of maternal and newborn health in Belgaum and Bagalkot in Karnataka over 2013–14. The mortality ratios and morbidity information can be used in planning interventions and monitoring indicators of effectiveness to inform policy and practice. Comprehensive regional epidemiologic data, such as that provided here, is essential to gauge improvements and challenges in maternal health, as well as track disparities found in rural areas.

## Introduction

Maternal and newborn mortality place significant burden on fragile health systems with 303,000 global maternal deaths annually [1]. Maternal mortality has decreased significantly across India with an estimated maternal mortality ratio (MMR) of 174 per 100,000 livebirths [139–217] in 2015, and an annual rate of reduction of 4.6% between 2000 and 2015[1]; however, progress has varied by region. Karnataka, a state in southern India, has better maternal and perinatal health indicators than much of the country with a MMR of 144, yet worse than neighbouring state of Kerala [2].

The major causes of maternal death and morbidity globally are haemorrhage, the hypertensive disorders of pregnancy, and sepsis [3]. In addition, chronic hypertension is strongly associated with pre-eclampsia and eclampsia, which in turn, may be responsible for maternal deaths related to other conditions: renal and hepatic disease, anaemia and systemic infections or sepsis [3, 4].

Comprehensive epidemiological data on maternal and perinatal mortality and morbidity are often missing from low- and middle-income countries (LMIC), particularly from rural areas. Improvements in health cannot be gauged without accurate population rates. In addition, rates are often underestimated without accurate vital registration centres. Existing health registration systems in India have been unable to reliably capture all pregnancies and outcomes, particularly in rural areas where most Indians live [5]. The inequities between health outcomes in urban and rural areas may be responsible for a difference in maternal mortality of 132%, as suggested by the Government of India National Family Health Survey (NFHS II, 1998–1999) [6].

This study was designed to assess baseline rates as the precursor to a community-based cluster randomized control trial (cRCT)–Community Level Interventions for Pre-eclampsia (CLIP) Trial (NCT01911494) [7]. The CLIP cRCT aims to reduce maternal and neonatal mortality and morbidity by the use of an evidence-based package of care for the community-level identification and emergency management of women at risk of developing eclampsia or pre-eclampsia. The trial is being conducted in India, Pakistan and Mozambique. The aims of this baseline assessment were, (1) to describe baseline demographics and maternal and newborn mortality prior to initiation of the CLIP cRCT in India, (2) to improve knowledge of population-level health across a broad geographic region of Karnataka, and (3) to identify the rates of the hypertensive disorders of pregnancy and other pregnancy complications in Belgaum and Bagalkot districts.

## Materials and Methods

### Setting

**Fig 1:**
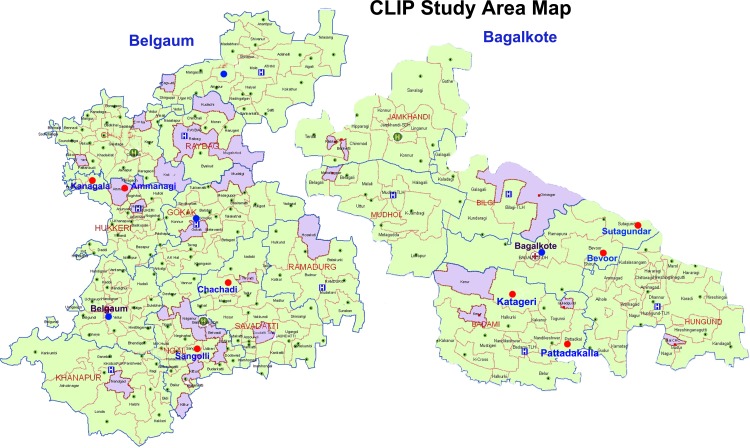
Study sites.

This study was conducted in eight distinct geographic regions in Karnataka State: four in Belgaum District and four in neighbouring Bagalkot District, with populations of 4,779,661and 1,889,752 in the 2011 census, respectively [8], and each with an estimated 500 annual births (Fig 1).

### Study design

The prospective population-based survey was adopted from the Maternal and Newborn Health (MNH) registry platform, developed by the Global Network for Women’s and Child Health Studies and in use in neighboring communities since 2009 [5]. Additional information relevant to the CLIP Trial was added in supplemental forms (S1–S10 Files). Data collection was incorporated within the framework of the health system utilizing existing health care staff where possible. All data was collected in the local language of Karnataka State–*Kannada*–and translated for analysis. As migration in pregnancy is common in this population, particularly in the first two pregnancies; the eligibility criteria included all women who were either a permanent resident of the area or non-resident who delivered in the study area. Women who migrated in pregnancy were followed postpartum to capture all pregnancy outcomes.

### Data collection

#### Step 1 –Assessment of eligibility through the register of married women of reproductive age

Data was collected from October 01, 2013 to October 31, 2014. The first step in the data collection process was identification of potentially eligible women. This was achieved through use of a ‘register of married women of reproductive age’ to identify eligible women, and to determine who was likely to conceive in the coming year. Community-based health workers, Accredited Social Health Activists (ASHA), were responsible for the identification of women likely to conceive through annual household visits. At this time ASHAs collected basic demographic information on couples, such as educational status, number of living children, age of last child, use of permanent family planning methods, sterility (primary, secondary, early menopause), and presence of an existing pregnancy. Basic analysis of this process was conducted for each region and validated against known trends for family size, number of couples, sterility rate, and birth rate. Women likely to conceive were visited periodically throughout the year, and when they missed their period, they were provided a pregnancy test to confirm the pregnancy.

#### Step 2 –Enrolment of eligible pregnancies and collection of pregnancy information

Confirmed pregnancies were then enrolled by the Registry Administrator (RA) and the first of three data collection forms were completed. Data were collected soon after pregnancy confirmation and enrollment was done in the home, sub-centre or primary health centre, and included information on past medical and obstetric history.

#### Step 3 –Collection of delivery information and pregnancy outcomes

Each enrolled pregnancy was followed-up at or immediately after delivery and then again at 42 days postpartum. Data were collected through review of medical records in the facility and interviews with birth attendants, providers and family members at the associated health centres and the woman’s home.

**Fig 2:**
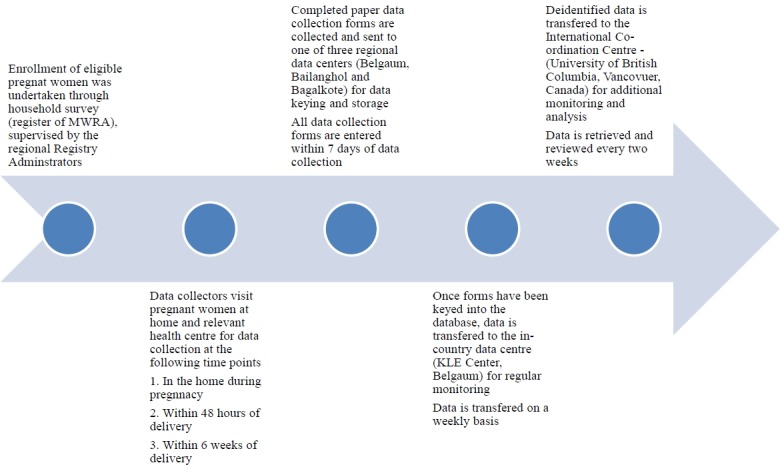
Data flow process.

Data forms were collected and transferred to the regional data centre (weekly), where forms were keyed into the database after confirming completeness. Rigorous data monitoring procedures were adopted from those employed by the Global Network MNH registry. Reliability and consistency of data collection procedures were ensured by monitoring visits and by reducing data entry errors, including re-keying of data (5% of all data forms). The accuracy of data entered was ensured by random chart review (5% of chart forms), and verification by the review of margins of error in reporting. Patient confidentiality was ensured by removal of all personal identifiers before data transfer. Furthermore, data integrity was maintained by use of password-protected data management systems. (For details of the data collection processes see Fig 2)

### Analysis

Descriptive statistics were completed using SAS and R. For continuous variables, either the mean and standard deviation, or the median and inter-quartile range is presented. For categorical variables, the number and percentage of responses is presented.

### Ethical approval

The study was approved by Institutional Ethics Committees (JNMC India and UBC Canada), Government of Karnataka and the Indian Council for Medical Research. Written informed consent was obtained from all participants. All participants retained one copy of the signed consent form for their records and an additional copy was kept at the research centre for study records. All consent forms and procedures were approved by both institutional ethics committees.

## Results

All pregnancies from October 01 2013 to October 31 2014 were included for analysis. In total, information was collected on 5,469 pregnancies, and 5,448 completed pregnancies with delivery outcomes. Very few women were lost to follow-up (n = 21, 0.38% of all pregnancies).

### Demographics

The average age of women in the cohort was 23.2 (+/- 3.3) years, they were enrolled early in pregnancy at 17.5–21.5 weeks gestation on average. Women were predominantly Hindu, with over 90% in each region, followed by approximately 5% Muslim. Many women reported being illiterate, with similar rates for men and women but variability between geographic regions. (Table 1)

**Table 1:** Population characteristics.

		Belgaum	Belgaum	Belgaum	Belgaum	Bagalkot	Bagalkot	Bagalkot	Bagalkot
		Ammangi	Kanagala	Chachadi	Sangolli	Katageri	Pattadakallu	Bevoor	Sutugundar
	Total								
Women	5,469	614	580	763	998	609	745	476	684
Average age	23.2+/-3.3	22.9+/-3.3	22.9+/-3.7	23.4+/-3.4	23.2+/-3.1	23.5+/-3.2	23+/-3.3	23.2+/-3.2	23+/-2.8
Gestational age at enrolment (weeks)	19.6+/-8.7	19.1+/-8.0	20+/-8.0	21.3+/-8.2	17.5+/-8.1	19.8+/-8.9	21.5+/-10	18.5+/-8.3	18.4+/-8.8
**Religion**									
Hindu	5137 (93.9%)	560 (91.2%)	535 (92.2%)	752 (98.6%)	886 (88.8%)	582 (95.6%)	719 (96.5%)	453 (95.2%)	650 (95%)
Muslim	299 (5.5%)	52 (8.5%)	42 (7.2%)	11 (1.4%)	95 (9.5%)	27 (4.4%)	20 (2.7%)	20 (4.2%)	32 (4.7%)
Other*	33 (%)	2 (%)	3 (0.5%)	0 (0%)	17 (%)	0 (0%)	6 (0.8%)	3 (%)	2 (%)
**Maternal level of education**									
Illiterate	1027 (18.8%)	35 (5.7%)	17 (2.9%)	284 (37.2%)	116 (11.6%)	103 (16.9%)	210 (28.2%)	92 (19.3%)	170 (24.9%)
Literate no school	122 (2.2%)	1 (0.2%)	6 (1%)	4 (0.5%)	11 (1.1%)	46 (7.6%)	22 (3%)	6 (1.3%)	26 (3.8%)
School	4319 (79%)	578 (94.1%)	557 (96%)	475 (62.3%)	871 (87.3%)	459 (75.5%)	513 (68.9%)	378 (79.4%)	488 (71.3%)
Don't know	1 (0%)	0 (0%)	0 (0%)	0 (0%)	0 (0%)	1 (0.2%)	0 (0%)	0 (0%)	0 (0%)
**Husband’s level of education**									
Illiterate	1004 (18.4%)	31 (5.0%)	19 (3.3%)	230 (30.1%)	169 (16.9%)	94 (15.5%)	175 (23.5%)	92 (19.3%)	194 (28.4%)
Literate no school	112 (2.0%)	0 (0.0%)	2 (0.3%)	7 (0.9%)	8 (0.8%)	25 (4.1%)	23 (3.1%)	3 (0.6%)	44 (6.5%)
School	4348 (79.6%)	583 (95.0%)	558 (96.4%)	526 (68.9%)	821 (82.3%)	489 (80.4%)	546 (73.4%)	381 (80.0%)	444 (65.1%)
Don't know	5 (0.1%)	0 (0%)	1 (0.2%)	0 (0%)	0 (0%)	1 (0.2%)	1 (0.1%)	0 (0%)	2 (0.3%)
**Poverty level**									
BLP card holder	4170 (76.2%)	481 (78.3%)	420 (72.4%)	587 (76.9%)	848 (85%)	443 (72.7%)	606 (81.3%)	333 (70%)	452 (66.1%)

*other religion includes those who self-identified as Christian, Jain, Sikh or other.

### Past medical and obstetric history

Past medical and obstetric history was self-reported for all pregnancies. Findings suggest a lower than expected rate of several past medical conditions: hypertension (1.1%), seizures (0.4%), diabetes (0.1%), tuberculosis (1%), and malaria (0.3%). There were low rates of past contraceptive use; the highest rate of use was 13.5% in one region. Hemoglobin was measured at enrollment or retrieved from a recent lab report. These results indicated a high rate of anemia, though some regions also had a high proportion of missing values. (Table 2)

**Table 2:** Past medical history.

		Belgaum	Belgaum	Belgaum	Belgaum	Bagalkot	Bagalkot	Bagalkot	Bagalkot
		Ammangi	Kanagala	Chachadi	Sangolli	Katageri	Pattadakallu	Bevoor	Sutugundar
	Total								
Women	5,469	614	580	763	998	609	745	476	684
Hypertension	59 (1.1%)	15 (2.4%)	14 (2.4%)	10 (1.3%)	6 (0.6%)	9 (1.5%)	3 (0.4%)	0(0%)	2 (0.3%)
Seizures	20 (0.4%)	3 (0.5%)	1 (0.2%)	6 (0.8%)	3 (0.3%)	3 (0.5%)	3 (0.4%)	0 (0%)	1 (0.1%)
Diabetes	7 (0.1%)	1 (0.2%)	3 (0.5%)	0 (0%)	1 (0.1%)	0 (0%)	1 (0.1%)	0 (0%)	1 (0.1%)
Tuberculosis	57 (1%)	7 (1.1%)	9 (1.6%)	6 (0.8%)	23 (2.3%)	8 (1.3%)	3 (0.4%)	0 (0%)	1 (0.1%)
Malarial medication use	18 (0.3%)	1 (0.2%)	3 (0.5%)	7 (0.9%)	1 (0.1%)	3 (0.5%)	0 (0%)	0 (0%)	3 (0.4%)
Contraceptive use	242 (4.6%)	20 (3.5%)	74 (13.5%)	18 (2.5%)	32 (3.3%)	42 (7%)	3 (0.4%)	33 (6.9%)	20 (3%)
Missing	192	43	33	38	40	13	18	0	7
Anaemia (Hemoglobin<11) ^⌂^	4569 (89%)	478 (81.4%)	510 (87.9%)	744 (98.8%)	921 (92.7%)	425 (88.7%)	589 (93.6%)	363 (79.4%)	539 (82.4%)
Anaemia—severe (Hemoglobin<7) ^⌂^	32 (0.6%)	4 (0.7%)	4 (0.7%)	1 (0.1%)	1 (0.1%)	6 (1.3%)	7 (1.1%)	2 (0.4%)	7 (1.1%)
Missing^⌂^	337	27	0	10	5	130	116	19	30

^⌂^ Information was collected at the time of enrolment.

Most women in the cohort were gravida 2; their previous pregnancy outcomes are reported in Table 3. Questions regarding complications in previous pregnancies were asked, in particular related to the hypertensive disorders of pregnancy as a condition leading to high rates of morbidity and mortality in India. The rate of previous hypertension in pregnancy was self-reported to be around 3%. Diabetes was also probed as an emerging illness in India affecting women in pregnancy, reported rates were very low. As an important indicator of health care utilization, the location of past delivery was asked. Results show distinct differences in delivery location between regions, this may be related to access to facilities; however, very few women report delivery outside facility, with only approximately 9% reporting home deliveries. (Table 3)

**Table 3:** Past obstetric history.

		Belgaum	Belgaum	Belgaum	Belgaum	Bagalkot	Bagalkot	Bagalkot	Bagalkot
		Ammangi	Kanagala	Chachadi	Sangolli	Katageri	Pattadakallu	Bevoor	Sutugundar
	Total	1	2	3	4	5	6	7	8
Women surveyed	5,469	614	580	763	998	609	745	476	684
Gravidity	2.1+/-1.1	2+/-1	2.1+/-1.2	2.1+/-1	1.9+/-1	2.1+/-1	2.1+/-1.2	1.9+/-1	2.2+/-1.1
Parity	1+/-1.1	1+/-1	1+/-1	1.1+/-1	0.9+/-1	1.1+/-1	1.1+/-1.1	1+/-1.5	1.2+/-1.2
**Outcomes of previous pregnancies**									
Miscarriages	242 (6.9%)	29 (7.5%)	56 (14.4%)	36 (7%)	31 (5.2%)	32 (7.7%)	19 (3.9%)	6 (2.2%)	33 (7.2%)
Medical termination of pregnancy	85 (2.4%)	13 (3.3%)	20 (5.2%)	17 (3.3%)	6 (1%)	14 (3.4%)	7 (1.4%)	2 (0.7%)	6 (1.3%)
Stillbirths	134 (3.8%)	4 (1%)	12 (3.1%)	21 (4.1%)	25 (4.2%)	19 (4.6%)	25 (5.1%)	7 (2.6%)	21 (4.6%)
Livebirths	3384 (96%)	378 (97.2%)	359 (92.5%)	496 (96.1%)	569 (96%)	395 (95.4%)	472 (96.1%)	269 (98.5%)	446 (96.7%)
Early neonatal deaths	101 (2.9%)	6 (1.5%)	12 (3.1%)	21 (4.1%)	17 (2.9%)	12 (2.9%)	18 (3.7%)	1 (0.4%)	14 (3%)
Late neonatal deaths	33 (0.9%)	0 (0%)	5 (1.3%)	3 (0.6%)	6 (1%)	10 (2.4%)	8 (1.6%)	0 (0%)	1 (0.2%)
**Complications in previous pregnancies**									
Hypertension	104 (3%)	9 (2.3%)	24 (6.2%)	18 (3.5%)	20 (3.4%)	16 (3.9%)	9 (1.8%)	2 (0.7%)	6 (1.3%)
Seizures	28 (0.8%)	2 (0.5%)	5 (1.3%)	5 (1%)	6 (1%)	3 (0.7%)	4 (0.8%)	1 (0.4%)	2 (0.4%)
Diabetes	11 (0.3%)	0 (0%)	4 (1%)	2 (0.4%)	1 (0.2%)	2 (0.5%)	2 (0.4%)	0 (0%)	0 (0%)
**Delivery location in previous pregnancy**									
Home	325 (9.2%)	18 (4.6%)	36 (9.3%)	50 (9.7%)	22 (3.7%)	37 (8.9%)	36 (7.3%)	32 (11.7%)	94 (20.4%)
Sub-centre	10 (0.3%)	0 (0%)	1 (0.3%)	0 (0%)	2 (0.3%)	3 (0.7%)	0 (0%)	3 (1.1%)	1 (0.2%)
Primary health centre	1420 (40.3%)	82 (21.1%)	49 (12.6%)	228 (44.2%)	337 (56.8%)	114 (27.5%)	208 (42.4%)	159 (58.2%)	243 (52.7%)
Government hospital	1019 (28.9%)	137 (35.2%)	157 (40.5%)	147 (28.5%)	166 (28%)	145 (35%)	166 (33.8%)	45 (16.5%)	56 (12.1%)
Private clinic	142 (4%)	27 (6.9%)	12 (3.1%)	24 (4.7%)	17 (2.9%)	17 (4.1%)	10 (2%)	17 (6.2%)	18 (3.9%)
Private hospital	600 (17%)	125 (32.1%)	133 (34.3%)	64 (12.4%)	49 (8.3%)	95 (22.9%)	70 (14.3%)	17 (6.2%)	47 (10.2%)
In transport	5 (0.1%)	0 (0%)	0 (0%)	2 (0.4%)	0 (0%)	3 (0.7%)	0 (0%)	0 (0%)	0 (0%)
Other	3 (0.1%)	0 (0%)	0 (0%)	1 (0.2%)	0 (0%)	0 (0%)	0 (0%)	0 (0%)	2 (0.4%)
Don't know	1 (0%)	0 (0%)	0 (0%)	0 (0%)	0 (0%)	0 (0%)	1 (0.2%)	0 (0%)	0 (0%)

### Care seeking in pregnancy

All women described care seeking practices in the current pregnancy–use of antenatal care services, delivery location, and birth attendant. Nearly all women reported some antenatal care utilization (98.9%); however, the number of visits differed between regions from an average of 2.7 (+/-1.4) to 7.6 (+/-2.6). Most women reported receiving tetanus vaccinations and blood pressure measured, as an indicator of regular antenatal care. Few women reported taking malaria medication (0.6%), yet many did report sleeping under a mosquito net ‘consistently’ (17.2%) or ‘occasionally’ (58.9%), as a method of malaria prophylaxis. Care at a higher level facility was not particularly common with the exception of two regions that estimated approximately 35% of women did seek care at higher level facility; this may be related to proximity of such services. (Table 4)

**Table 4:** Care seeking in pregnancy.

		Belgaum	Belgaum	Belgaum	Belgaum	Bagalkot	Bagalkot	Bagalkot	Bagalkot
		Ammangi	Kanagala	Chachadi	Sangolli	Katageri	Pattadakallu	Bevoor	Sutugundar
	Total	1	2	3	4	5	6	7	8
Pregnancies	5469	614	580	763	998	609	745	476	684
**Care-seeking in this pregnancy**									
Women with antenatal care	5386 (98.9%)	614 (100%)	575 (99.1%)	743 (97.4%)	995 (99.7%)	604 (99.2%)	723 (97.3%)	449 (98.2%)	683 (99.9%)
Times such care was sought	4.5+/-2	7.6+/-2.6	5.2+/-1.8	4.2+/-1.3	4.5+/-1.1	4.6+/-1.5	2.7+/-1.4	3.9+/-1	3.6+/-1.3
Higher level facility-based care	618 (11.3%)	220 (35.8%)	15 (2.6%)	41 (5.4%)	9 (0.9%)	217 (35.6%)	22 (3%)	27 (5.9%)	67 (9.8%)
**Other antenatal care**									
Tetanus toxoid vaccine	5268 (96.7%)	570 (92.8%)	547 (94.3%)	738 (96.7%)	972 (97.4%)	589 (96.7%)	722 (97.2%)	454 (99.3%)	676 (98.8%)
Blood pressure measured	5293 (97.2%)	577 (94%)	552 (95.2%)	740 (97%)	992 (99.4%)	595 (97.7%)	716 (96.4%)	453 (99.1%)	668 (97.7%)
Malaria medication	33 (0.6%)	6 (1%)	4 (0.7%)	2 (0.3%)	2 (0.2%)	3 (0.5%)	5 (0.7%)	5 (1.1%)	6 (0.9%)
Consistently slept under mosquito net	938 (17.2%)	6 (1%)	14 (2.4%)	111 (14.5%)	450 (45.1%)	162 (26.6%)	10 (1.3%)	15 (3.3%)	170 (24.9%)
Occasionally slept under mosquito net	3209 (58.9%)	208 (33.9%)	544 (93.8%)	483 (63.3%)	540 (54.1%)	359 (58.9%)	377 (50.7%)	419 (91.7%)	279 (40.8%)
Never slept under mosquito net	1303 (23.9%)	400 (65.1%)	22 (3.8%)	169 (22.1%)	8 (0.8%)	88 (14.4%)	356 (47.9%)	24 (5.3%)	236 (34.5%)

Nearly all deliveries occurred in facility, split between hospitals and clinics/primary health centres. The remaining small number of women delivered at home, less than 5% overall. Efforts to increase institutional deliveries have been successful in these regions where nearly all women delivered in facility with a skilled birth attendant: obstetrician (39.8%), doctor (21.9%), nurse or midwife (34.6%). (Table 5)

**Table 5:** Delivery outcomes.

		Belgaum	Belgaum	Belgaum	Belgaum	Bagalkot	Bagalkot	Bagalkot	Bagalkot
		Ammangi	Kanagala	Chachadi	Sangolli	Katageri	Pattadakallu	Bevoor	Sutugundar
	Total	1	2	3	4	5	6	7	8
Deliveries	5448	614	580	763	998	609	743	457	684
Delivery location*									
Hospital	2436 (48.8%)	388 (70.7%)	404 (80.5%)	237 (34.1%)	253 (27.5%)	327 (58%)	321 (45.9%)	246 (56.7%)	260 (41.2%)
Clinic/primary health centre	2356 (47.2%)	154 (28.1%)	87 (17.3%)	435 (62.7%)	659 (71.6%)	193 (34.2%)	342 (48.9%)	152 (35%)	334 (52.9%)
Home	191 (3.8%)	7 (1.3%)	9 (1.8%)	20 (2.9%)	7 (0.8%)	41 (7.3%)	36 (5.1%)	33 (7.6%)	38 (6%)
Other	13 (0.3%)	0 (0%)	2 (0.4%)	2 (0.3%)	2 (0.2%)	3 (0.5%)	1 (0.1%)	3 (0.7%)	0 (0%)
Birth attendant*									
Obstetrician	1988 (39.8%)	371 (67.6%)	266 (53%)	218 (31.4%)	255 (27.7%)	232 (41.1%)	258 (36.9%)	196 (45.2%)	192 (30.4%)
Doctor	1092 (21.9%)	156 (28.4%)	6 (1.2%)	28 (4%)	444 (48.2%)	63 (11.2%)	161 (23%)	151 (34.8%)	83 (13.2%)
Nurse/ midwife	1727 (34.6%)	16 (2.9%)	222 (44.2%)	424 (61.1%)	218 (23.7%)	226 (40.1%)	246 (35.1%)	53 (12.2%)	322 (51%)
Traditional birth attendant	19 (0.4%)	0 (0%)	0 (0%)	0 (0%)	0 (0%)	1 (0.2%)	12 (1.7%)	4 (0.9%)	2 (0.3%)
Family	135 (2.7%)	4 (0.7%)	7 (1.4%)	18 (2.6%)	0 (0%)	39 (6.9%)	17 (2.4%)	25 (5.8%)	25 (4%)
Self	21 (0.4%)	0 (0%)	0 (0%)	3 (0.4%)	3 (0.3%)	3 (0.5%)	6 (0.9%)	5 (1.2%)	1 (0.2%)
Other	13 (0.3%)	2 (0.4%)	1 (0.2%)	3 (0.4%)	1 (0.1%)	0 (0%)	0 (0%)	0 (0%)	6 (1%)
**Mode of delivery**									
Vaginal	4306 (79%)	441 (71.8%)	376 (64.8%)	647 (84.8%)	855 (85.7%)	456 (74.9%)	622 (83.7%)	352 (77%)	557 (81.4%)
Vaginal without forceps/ vacuum	3758 (69%)	418 (68.1%)	351 (60.5%)	477 (62.5%)	823 (82.5%)	449 (73.7%)	464 (62.4%)	314 (68.7%)	462 (67.5%)
Vaginal with forceps/ vacuum	549 (10.1%)	23 (3.7%)	25 (4.3%)	170 (22.3%)	32 (3.2%)	7 (1.1%)	158 (21.3%)	38 (8.3%)	96 (14%)
C-section	692 (12.7%)	109 (17.8%)	126 (21.7%)	47 (6.2%)	67 (6.7%)	108 (17.7%)	78 (10.5%)	82 (17.9%)	75 (11%)
Miscarriage	292 (5.4%)	44 (7.2%)	49 (8.4%)	44 (5.8%)	60 (6%)	23 (3.8%)	21 (2.8%)	16 (3.5%)	35 (5.1%)
Medical termination of pregnancy	158 (2.9%)	20 (3.3%)	29 (5%)	24 (3.1%)	16 (1.6%)	22 (3.6%)	22 (3%)	7 (1.5%)	18 (2.6%)

*does not include miscarriages of medical terminations of pregnancy.

### Pregnancy outcomes

**Fig 3:**
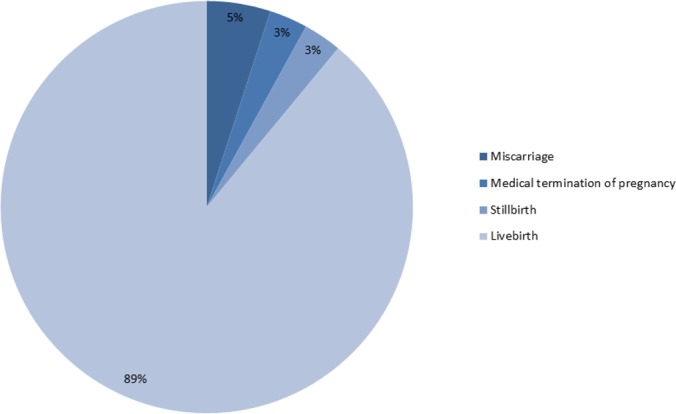
Pregnancy outcomes.

The results of pregnancies in this cohort indicate a rate of miscarriage of 5.4%, medical termination of pregnancy of 2.9%, stillbirth of 2.6%, and livebirths of 89.1% (Fig 3). Pregnancy losses at less than 20 weeks were classified as miscarriages or medical terminations of pregnancy, while losses at 20 weeks or greater were defined as stillbirths. The average gestational age at delivery was 37.6 (+/- 6.5) weeks. One region had a substantially lower average gestational age at delivery (34.9 weeks); however, the rate of preterm birth did not appear to be greater. Preterm deliveries less than 37 weeks accounted for roughly 16% of all deliveries, with early preterm (<34 weeks) accounted for half (8% of all deliveries). Results indicate no sex differential in infants with 50.9% male (95% confidence interval [49.2, 52.7]) and 49.1% female.

**Fig 4:**
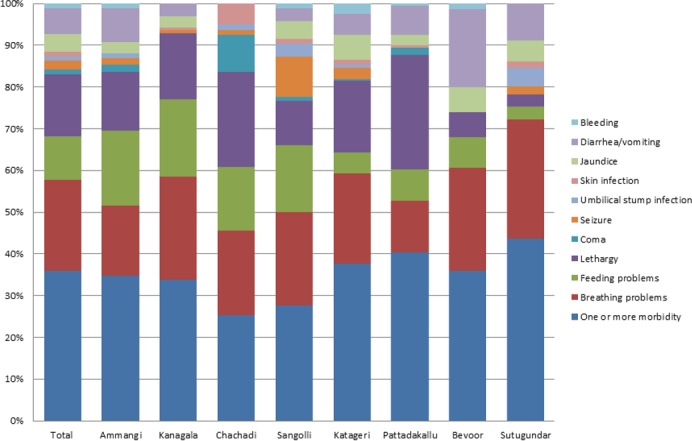
Neonatal morbidities.

A range of neonatal morbidities were assessed for all infants up to 28 days of age. The most common neonatal complications amongst this population were breathing difficulties (4.1%), lethargy (2.8%), and feeding problems (2%) (Fig 4). In total, 5.9% of infants suffered one or more morbidities. The mortality ratios amongst infants were: neonatal mortality ratio of 25 per 1,000 livebirths, and a perinatal mortality ratio of 50 per 1,000 livebirths. In addition, a total of 143 pregnancies (2.6%) resulted in a stillbirth.

Numerous pregnant women in this cohort suffered one or more morbidities (2.9%). The most commonly reported morbidities were blood transfusion (1.9%) and suspected sepsis (0.8%). Suspected sepsis was defined as fever with one or more symptoms–headache and stiff neck, cough and shortness of breath, abdominal pain and uterine tenderness, flank pain, or foul smelling discharge. There were five maternal deaths throughout the study period. (Table 6)

**Table 6:** Maternal and perinatal morbidities and mortalities.

		Belgaum	Belgaum	Belgaum	Belgaum	Bagalkot	Bagalkot	Bagalkot	Bagalkot
		Ammangi	Kanagala	Chachadi	Sangolli	Katageri	Pattadakallu	Bevoor	Sutugundar
	Total	1	2	3	4	5	6	7	8
Pregnancies	5469	614	580	763	998	609	745	476	684
Deliveries	5448	614	580	763	998	609	743	457	684
Infants	5488	623	584	768	1003	611	749	462	688
Multiple pregnancies	39 (0.8%)	8 (1.5%)	4 (0.8%)	5 (0.7%)	5 (0.5%)	2 (0.4%)	6 (0.9%)	5 (1.2%)	4 (0.6%)
Gestational age at delivery (weeks)	37.6+/-6.5	34.9+/-11.8	36.4+/-9.3	36.5+/-8.7	38.7+/-3.9	38.5+/-2.8	38.2+/-3.1	38.3+/-2.8	38.7+/-2.7
Preterm deliveries <34 weeks	448 (8.2%)	54 (8.8%)	57 (9.8%)	60 (7.9%)	47 (4.7%)	53 (8.7%)	70 (9.4%)	32 (7%)	75 (11%)
Preterm deliveries <37 weeks	879 (16.1%)	91 (14.8%)	100 (17.2%)	119 (15.6%)	97 (9.7%)	109 (17.9%)	159 (21.4%)	84 (18.4%)	120 (17.5%)
**Birth outcomes** *(of total pregnancies)*									
Miscarriage	294 (5.4%)	45 (7.3%)	49 (8.4%)	44 (5.8%)	61 (6.1%)	23 (3.8%)	21 (2.8%)	16 (3.5%)	35 (5.1%)
Medical termination of pregnancy	158 (2.9%)	20 (3.3%)	29 (5%)	24 (3.1%)	16 (1.6%)	22 (3.6%)	22 (3%)	7 (1.5%)	18 (2.6%)
Stillbirth	143 (2.6%)	20 (3.3%)	9 (1.6%)	20 (2.6%)	17 (1.7%)	19 (3.1%)	25 (3.4%)	19 (4.2%)	14 (2%)
Livebirth	4852 (89.1%)	529 (86.2%)	493 (85%)	674 (88.3%)	904 (90.6%)	545 (89.5%)	675 (90.8%)	415 (90.8%)	617 (90.2%)
**Neonatal outcomes**									
Male	2550 (50.9%)	295 (53.5%)	244 (48.6%)	348 (50.0%)	465 (50.4%)	298 (52.8%)	352 (50.2%)	226 (51.8%)	322 (50.9%)
Female	2455 (49.1%)	256 (46.5%)	258 (51.4%)	348 (50.0%)	458 (49.6%)	266 (47.2%)	349 (49.8%)	210 (48.2%)	310 (49.1%)
Don't know	6 (0.1%)	0 (0%)	0 (0%)	1 (0.1%)	2 (0.2%)	2 (0.4%)	1 (0.1%)	0 (0%)	0 (0%)
Birthweight (g)	2765.3+/-474.6	2847.9+/-554.4	2731.2+/-476.8	2659+/-472	2805.2+/-432.9	2774.8+/-456.6	2803.4+/-431.7	2746.1+/-489.4	2742.9+/-487.6
Birthweight <2500g	809 (16.4%)	68 (12.7%)	83 (16.5%)	150 (21.7%)	132 (14.3%)	112 (20.4%)	73 (10.7%)	64 (14.8%)	127 (20.7%)
Birth injury	23 (0.5%)	9 (1.7%)	2 (0.4%)	0 (0%)	2 (0.2%)	2 (0.4%)	2 (0.3%)	4 (1%)	2 (0.3%)
Congenital anomaly	19 (0.3%)	2 (0.3%)	1 (0.2%)	4 (0.5%)	4 (0.4%)	0 (0%)	1 (0.1%)	0 (0%)	7 (1%)
One or more morbidity	322 (6.8%)	33 (6.4%)	53 (11%)	13 (2%)	23 (2.6%)	83 (15.6%)	43 (6.5%)	22 (5.4%)	52 (8.8%)
Breathing problems	226 (4.1%)	19 (3.1%)	47 (8.1%)	12 (1.6%)	21 (2.1%)	55 (9%)	15 (2%)	17 (3.7%)	40 (5.8%)
Feeding problems	110 (2%)	20 (3.3%)	35 (6%)	9 (1.2%)	15 (1.5%)	13 (2.1%)	9 (1.2%)	5 (1.1%)	4 (0.6%)
Lethargy	154 (2.8%)	16 (2.6%)	30 (5.2%)	14 (1.8%)	10 (1%)	43 (7.1%)	33 (4.4%)	4 (0.9%)	4 (0.6%)
Coma	11 (0.2%)	2 (0.3%)	0 (0%)	5 (0.7%)	1 (0.1%)	1 (0.2%)	2 (0.3%)	0 (0%)	0 (0%)
Seizure	23 (0.4%)	2 (0.3%)	1 (0.2%)	1 (0.1%)	9 (0.9%)	7 (1.1%)	0 (0%)	0 (0%)	3 (0.4%)
Umbilical stump infection	13 (0.2%)	1 (0.2%)	0 (0%)	1 (0.1%)	3 (0.3%)	2 (0.3%)	0 (0%)	0 (0%)	6 (0.9%)
Skin infection	11 (0.2%)	0 (0%)	1 (0.2%)	3 (0.4%)	1 (0.1%)	3 (0.5%)	1 (0.1%)	0 (0%)	2 (0.3%)
Jaundice	41 (0.8%)	3 (0.5%)	5 (0.9%)	0 (0%)	4 (0.4%)	15 (2.5%)	3 (0.4%)	4 (0.9%)	7 (1%)
Diarrhea/vomiting	64 (1.2%)	9 (1.5%)	6 (1%)	0 (0%)	3 (0.3%)	13 (2.1%)	8 (1.1%)	13 (2.8%)	12 (1.8%)
Bleeding	10 (0.2%)	1 (0.2%)	0 (0%)	0 (0%)	1 (0.1%)	6 (1%)	1 (0.1%)	1 (0.2%)	0 (0%)
Stillbirth	143 (2.6%)	20 (3.3%)	9 (1.6%)	20 (2.6%)	17 (1.7%)	19 (3.1%)	25 (3.4%)	19 (4.2%)	14 (2%)
Early neonatal death	101 (2.1%)	9 (1.7%)	10 (2%)	12 (1.8%)	19 (2.1%)	10 (1.8%)	12 (1.8%)	10 (2.4%)	19 (3.1%)
Late neonatal death	19 (0.4%)	2 (0.4%)	0 (0%)	2 (0.3%)	5 (0.6%)	1 (0.2%)	2 (0.3%)	1 (0.2%)	6 (1%)
**Maternal morbidities**									
One or more morbidity	160 (3.3%)	14 (2.7%)	24 (4.9%)	17 (2.5%)	21 (2.3%)	28 (5.1%)	11 (1.6%)	13 (3.1%)	32 (5.2%)
Blood transfusion	102 (1.9%)	16 (2.6%)	19 (3.3%)	7 (0.9%)	7 (0.7%)	13 (2.1%)	10 (1.3%)	10 (2.2%)	20 (2.9%)
Hysterectomy	5 (0.1%)	0 (0%)	0 (0%)	1 (0.1%)	1 (0.1%)	1 (0.2%)	1 (0.1%)	0 (0%)	1 (0.1%)
Antepartum haemorrhage	28 (0.5%)	0 (0%)	3 (0.5%)	9 (1.2%)	4 (0.4%)	4 (0.7%)	3 (0.4%)	0 (0%)	5 (0.7%)
Coma	3 (0.1%)	1 (0.2%)	1 (0.2%)	0 (0%)	0 (0%)	0 (0%)	0 (0%)	0 (0%)	1 (0.1%)
Failure to form clots	17 (0.3%)	0 (0%)	0 (0%)	2 (0.3%)	1 (0.1%)	1 (0.2%)	0 (0%)	0 (0%)	13 (1.9%)
Fever and symptom of sepsis	41 (0.8%)	2 (0.3%)	1 (0.2%)	8 (1%)	12 (1.2%)	14 (2.3%)	0 (0%)	0 (0%)	4 (0.6%)
Bimanual uterine compression	4 (0.1%)	0 (0%)	0 (0%)	0 (0%)	0 (0%)	0 (0%)	0 (0%)	3 (0.7%)	1 (0.1%)
Brace sutures	3 (0.1%)	0 (0%)	0 (0%)	0 (0%)	0 (0%)	0 (0%)	0 (0%)	3 (0.7%)	0 (0%)
CPR	2 (0%)	0 (0%)	1 (0.2%)	0 (0%)	0 (0%)	0 (0%)	0 (0%)	0 (0%)	1 (0.1%)
Dialysis	1 (0%)	0 (0%)	0 (0%)	1 (0.1%)	0 (0%)	0 (0%)	0 (0%)	0 (0%)	0 (0%)
Internal iliac artery ligation/ devascularisation	3 (0.1%)	0 (0%)	0 (0%)	0 (0%)	0 (0%)	0 (0%)	0 (0%)	3 (0.7%)	0 (0%)
Mechanical ventilation	2 (0%)	0 (0%)	0 (0%)	0 (0%)	0 (0%)	0 (0%)	0 (0%)	2 (0.4%)	0 (0%)
Seizure	15 (0.3%)	2 (0.3%)	6 (1%)	1 (0.1%)	1 (0.1%)	2 (0.3%)	1 (0.1%)	0 (0%)	2 (0.3%)
Stroke	1 (0%)	0 (0%)	1 (0.2%)	0 (0%)	0 (0%)	0 (0%)	0 (0%)	0 (0%)	0 (0%)
Severe postpartum hemorrhage	28 (0.5%)	2 (0.3%)	1 (0.2%)	2 (0.3%)	1 (0.1%)	6 (1%)	4 (0.5%)	1 (0.2%)	11 (1.6%)
Maternal deaths	5 (0.1%)	0 (0%)	1 (0.2%)	2 (0.3%)	0 (0%)	0 (0%)	0 (0%)	0 (0%)	2 (0.3%)

## Discussion

In this study, delivery at home was reported for only 3.8% deliveries, much less than the reported rate by the District Level Household and Facility Survey (DLHS) of 13.4% [9]. Similarly, rates of institutional deliveries were 96.0%, much higher than reported in 85.8% rural Karnataka and other studies [10]. These differences could be due to the variability in time demographic composition studied in these surveys.

Consistent with the information from the 2011 census data for Karnataka, higher number of male infants were reported, although the difference is not statistically significant [8]. The disparity has been demonstrated in rural and urban areas in Belgaum and Bagalkot, although in rural Belgaum the sex-differential at birth is greater (rural: 37,288 males vs. 32,915 females). The sex ratio in Karnataka is consistent with long-standing cultural beliefs regarding the perceived higher value of male children [8].

In Karnataka, the establishment of the National Rural Health Mission (NRHM)‘ *Janani Suraksha Vahini*’ (a programme in which special vehicles are designated for obstetric emergency transport) has improved access to facilities in pregnancy [11]. Despite the increase in institutional births, the proportion of livebirths in this study (89%) was lower than the comparable estimates of 95.5% in rural Karnataka [9]. A notable finding in this study is the rate of stillbirth; this was found to be 28.6 per 1,000 births whereby stillbirth was defined with an assumed gestational age of 20 weeks or more. In a recent study, the rate of national average rate of stillbirth was found to be 23.0 per 1,000 births, however the gestational age used to define stillbirth was 28 weeks or more, thus only accounting for late fetal deaths[12]. The gestational age cutoff of 20 weeks or more gestation used to define stillbirth in this study includes numbers that include early fetal deaths, which are not accounted for in similar studies. Globally, there has been a reduction in the stillbirth rate consequent to increases in facility births and caesarean section rates; however, stillbirth rates are high, indicating that quality of care and timely access to it continues to challenge the health system [13]. Increasing availability of obstetricians and emergency obstetric care in rural Karnataka may help to reduce the proportion of stillbirths that occur intrapartum, a majority of which are preventable [12].

The NRHM has been successful in strengthening the infrastructure of primary health care, there is, however, a large gap in the delivery of obstetric emergency services in Karnataka, where only 36.6% of the community health centres have an obstetrician, and only 23.1% community health centres offer caesearan-section services (DLHS-4) [9]. This disparity in health services is greatest in rural areas [14]. Furthermore, higher antenatal care coverage has been associated with lower rates of antepartum stillbirth [12]. In this study, a vast majority (98.9%) of women reported at least one antenatal visit, which is higher than other studies in the state (93.4%) and 93.1% reported for rural Karnataka in the 2012–2013 DLHS-4 [9, 10]. Although the rates of antenatal reach in this study seem to be high, the frequency of visits per woman appear to be teetering on the edge of the World Health Organization’s (WHO) recommended four antenatal visits [15], ranging between an average of 2.7–7.6. The four visit antenatal care model for low-risk populations, suggested by the WHO, is currently under review [16]. There is a push to optimize the timing of delivery and a focus on the quality of visits over frequency. Strengthening the quality of antenatal care to encompass family planning is essential to decreasing adverse maternal and neonatal outcomes.

Studies among marginalized groups in the state of Karnataka, which remains among the top ten states in India with a high proportion of scheduled castes, and scheduled tribes, indicate that this socioeconomically vulnerable population show poor utilization of family planning and maternal health services [8, 10]. This population level study did not garner information on differential social classes, although does bring insight into the care seeking behavior in economically disadvantaged rural populations.

During the course of antenatal care, 97.2% of women reported having had at least one blood pressure measurement, much higher than the 83.2% reported elsewhere [9]. Furthermore, 96.7% of women report receiving at least one administration of tetanus toxoid vaccine, which is higher than the state-level estimate of 90.1% in rural areas [9]. Prevalence of anaemia (<11g/dL) and severe aneamia (<7g/dL) in pregnancy were much higher in this study (89% and 0.6%, respectively) than state estimates of 62.5% and 7.1%, respectively; however, the large number of missing values for anaemia make it difficult to establish reliable estimates. The rate of blood transfusion (1.9%) may be linked with these high rates of anemia.

The data shared in this paper provides critical insight to understanding the health profile of the rural women in two districts of Karnataka state. Inequalities in maternal health are shaped by contextual as well as individual factors, particularly urban-rural differences. This paper provides data on the socio-demographic characteristics of the marginalized rural population, and furthermore provides information on range of individual level factors including morbidities. There exists a paucity of information on maternal morbidities from LMIC, despite the belief that for every maternal death at least 20 women experience a maternal morbidity [3]. Comparable regional estimates of maternal morbidities captured in this study are unavailable; however, in the DLHS-4 survey indicates 36.1% of women report pregnancy complications, 26.1% of women reported delivery complications, and 13.1% post-delivery complications [9].

It is pertinent to mention that findings from this population level study, such as the higher than national rates of stillbirth, may accurately reflect true prevalence and maybe more comprehensive than census data. It is suggested that globally, less than 5% of neonatal deaths and even fewer stillbirths (especially intrapartum stillbirths) are recorded or made available in hospital and national information systems, particularly in rural areas [12]. In addition, national vital registration systems can fail to record up to 50% of maternal deaths due to errors of misclassification resulting from errors in medical reporting, certifications, and challenges in applying the ICD-code [1]. In the absence of reliable clinical definitions, audit processes and data information systems in facilities, estimates offered by this study can be instrumental in monitoring and planning of health interventions.

This study is not free from the limitations of self-reported data, and may be susceptible to social desirability bias and inaccurate recall; however, the use of prospective data collection reduces the inaccuracies associated with recall and reporting. Early registration of pregnant women allowed for capturing information on pregnancy outcomes at earlier gestational ages including estimates of miscarriages and medical terminations of pregnancy. Furthermore, the systematic and close follow-up of pregnancies ensured that few women were lost to follow-up [17]. Close oversight and monitoring allowed the capture of most pregnancy outcomes, in spite of the very common practice of migration in pregnancy to their mothers’ home.

Since 2005, the NRHM has helped make substantial improvements to maternal and newborn health in rural India by investments in primary health infrastructure increases in institutional deliveries by programs such as ‘*Janani Suraksha Yojana***’**, and by the extension of community-based health activists ASHAs [11].Despite these strides, India is ranked first for stillbirth and neonatal deaths in 2015, and contributes to the highest proportion of maternal deaths in the world [1, 12].

Population-level estimates, as offered in this study, are essential to focus interventions and policies to improve maternal and newborn health in rural areas such as CLIP trial India, which not only increases the frequency of ante-natal visits but also the quality of care. This study provides vital statistics that can be used to guide maternal health interventions, to address preventable morbidities, including addressing effective ante-natal care, strengthening emergency obstetric care and contraceptive uptake in communities.

## Conclusions

This population level study establishes baseline rates of maternal and newborn health in Belgaum and Bagalkot districts in Karnataka over 2013–14, and aids in improving knowledge of population-level health. To monitor indicators of effectiveness of national programmes for improving maternal and newborn health such as the National Rural Health Mission, this information is valuable and can inform practice and policy.

## Supporting Information

S1 File Maintenance of Eligible Couple Register. (PDF)

S2 File Screening Log. (PDF)

S3 File Enrollment Form. (PDF)

S4 File Enrollment Form Kannada. (PDF)

S5 File Supplemental Enrollment Form. (PDF)

S6 File Perinatal Form. (PDF)

S7 File Supplemental Perinatal Form. (PDF)

S8 File Follow-up Form. (PDF)

S9 File Follow-up Form (Kannada). (PDF)

S10 File Supplemental Follow-up Form. (PDF)

## Abbreviations

LMIC: Low and middle-income Country
CLIP: Community Level Interventions for Pre-eclampsia
cRCT: cluster-randomised control trial
ASHA: Accredited Social Health Activist
GA: gestational age
BPL: Below Poverty Line
PHC: primary health centre
MNH: maternal newborn health
RA: registry administrator
NRHM: National Rural Health Mission
MDG: Millennium Development Goal
