# Supplementary material for: Maternal and Newborn Health in Karnataka State, India: The Community Level Interventions for Pre-Eclampsia (CLIP) Trial’s Baseline Study Results
Source: PLoS One. 2017 Jan 20;12(1):e0166623. doi: 10.1371/journal.pone.0166623 (PMC5249209; doi:10.1371/journal.pone.0166623)
Supplement: S1 File — (PDF) [file pone.0166623.s001.pdf]

Maintenance of Eligible Couple Register – Guidelines

Introduction:

The primary purpose of Maternal Newborn Health (MNH) Registry is to quantify and understand the trends in primary outcomes in defined geographic areas in order to provide population based statistics on Stillbirth, Neonatal and Maternal Mortality and to know the causes and to take remedial action. To accomplish this it is essential to Register and Track all pregnant women till 42 days end of pregnancy. The following method is developed and adopted for enrolling Married Women of Reproductive Age (MWRA) who are the permanent residents of the study area.

Guidelines:-

1. Every year a survey has to be conducted during the month of December and Analysis has to be done by visiting all the houses coming under Anganwadi area or under an ASHA worker's area and registering all the details of the newly married women (eligible couple) belonging to the age group of 15 years to 49 years.
2. Under the leadership of Maternal and Newborn Health Registry Administrator (Medical Officer) the ASHA worker or (ANGANAWADI WORKER) with the help of Auxiliary Nurse Midwife (ANM) has to note down all the details of the eligible couples, clearly and legibly

Section - 1: **Population of the area.**

I. To visit every house and note down the number of people living in that house and also the number of eligible couples in that house.
II. To note down without fail the number of people living in the house in houses which do not have eligible couples.
III. To exclude the people who are temporarily residing in the house (Eg: married daughter, relatives, etc.)

Section - 2: Information about the Eligible Couple:

General Information:-

I. EC No. 1, 2, 3, 4 ----------- should be registered serially starting from the left part of the region.
II. Name: To record the names of the woman and her husband separately and clearly.
III. Address: To record the husband's name and address of her mother's house along with the telephone number clearly and completely
IV. Religion and Caste: Related information should be noted down clearly and encircle it. If there is intercaste marriage to encircle the caste and religion of the husband.

Specific Information:

1. Date of registration : To note down the date of survey of house of the eligible couple.
2. Age : Note down the (completed years) age of husband and wife.
3. Educational status : Note down the level of education of the eligible couple.
4. Number of alive children : Note down the number of live male and female children separately.
5. Age of last Child : Note down the completed years and months separately.
6. Whether they have adopted permanent method of Sterilization :
    1. Female: Tubectomy, Male: Vasectomy (NSV).
       If done – 1, Not done - 2.
    2. Do not consider the temporary methods of sterilization.
7. Sterility :
    1. Primary sterility: Not pregnant even after 5 years of married life without the use of family planning methods.
    2. Secondary sterility: Not being pregnant for 5 yrs after the first pregnancy without the use of any family planning methods.
    3. Early menopause: Women aged 40-49 years regular menstrual cycles and leading normal married life who don't get menstrual cycles / bleeding for more than a year.
8. Whether woman is currently pregnant?
    1. If Women is pregnant – denote 1, if not pregnant – denote 2, To note down whether the woman (eligible couple) is pregnant with the relevant signs even if the woman has gone to her mothers house.
    2. To note down in a separate register if there are any pregnant women (daughter or any other relative during the survey of the house.

<u>Section 3:</u> Eligible Couple Analysis

1. Register the year of survey – **I**

2. Enter population of every ANGANAWADI or ASHA area- **II**

3. Enter the total number of eligible couples of every ANGANWADI Area – **III**

4. Enter the number of EC who have adopted permanent methods of sterilization – **IV**

5. Enter number of EC with primary sterility, secondary sterility, early menopause –**V,VI,VII**

6. Number of current pregnant women (EC or daughter in law of that house) –**VIII**

7. Number of EC who may not conceive – **IX= (IV+V+VI+VII+VIII)**

8. Number of EC likely to conceive in the current year- **X=(III-IX)**

Section – 1
Regional Population

Year 2013

House No.	EC No.	No. of Members	House No.	EC No.	No. of Members	House No.	EC No.	No. of Members	House No.	EC No.	No. of Members	House No.	EC No.	No. of Members
1			25			49			73			97		
2			26			50			74			98		
3			27			51			75			99		
4			28			52			76			100		
5			29			53			77			101		
6			30			54			78			102		
7			31			55			79			103		
8			32			56			80			104		
9			33			57			81			105		
10			34			58			82			106		
11			35			59			83			107		
12			36			60			84			108		
13			37			61			85			109		
14			38			62			86			110		
15			39			63			87			111		
16			40			64			88			112		
17			41			65			89			113		
18			42			66			90			114		
19			43			67			91			115		
20			44			68			92			Total:		
21			45			69			93			Number of Houses:_____		
22			46			70			94			Number of Members:___		
23			47			71			95			Number of EC:_____		
24			48			72			96					

Section – 2
Information of Eligible Couple

EC No:______________ EC Name: Wife:_______________________________________ Husband:_______________________________

Complete Address & Phone Number: 1. Husband's Address / House:____
____ Phone Number:______________________________

2. Mother's Address / House:____
____Phone Number:______________________________

Religion (encircle): Hindu/Muslim/Christian/Sikh/Jain/Others Caste: (encircle): SC/ST/Others

Year	Date of registration	Age (Completed Years)		Educational Status		No. of living children		Age of last child		Whether adopted permanent methods of sterilization? Yes – 1, No – 2		Sterility Primary-1 Secondary -2 Early Menopause - 3	Whether woman pregnant now? Yes – 1 No - 2	Remarks
		Husband	Wife	Husband	Wife	Male	Female	Years	Months	Husband	Wife			
1	2	3	4	5	6	7	8	9	10	11	12	13	14	15
2013														
2014														
2015														

Section – 3
Eligible Couple Analysis

Year	Population	Total No. of EC	Number of EC who cannot conceive						No. of EC likely to conceive	Remarks
			No. of couples using permanent sterilization methods	Sterility			No. of current pregnant women	Total (IV+V+VI+VII+VIII)		
				Primary	Secondary	Early Menopause				
I	II	III	IV	V	VI	VII	VIII	IX	X	XI
2013										
2014										
2015										
