# Supplementary material for: Maternal and Newborn Health in Karnataka State, India: The Community Level Interventions for Pre-Eclampsia (CLIP) Trial’s Baseline Study Results
Source: PLoS One. 2017 Jan 20;12(1):e0166623. doi: 10.1371/journal.pone.0166623 (PMC5249209; doi:10.1371/journal.pone.0166623)
Supplement: S2 File — (PDF) [file pone.0166623.s002.pdf]

| University of British Columbia<br>KLE University's JNMC & SNMC | Maternal Newborn Health Registry<br>**SCREENING LOG** | **MN00** |
|---|---|---|
| **Page** 1 of 1 | | **Version 2.0**<br>**July 16, 2013** |

This registry should be maintained by the Registry Administrator for each study cluster. All pregnant women who are residents of the study cluster or who deliver in a facility or home within the cluster should be registered as early as possible during pregnancy. The registry should be entered in the DMS and transmitted to RTI on a weekly basis.

| **1. National or Ministry of Health ID # if known or 9999**<br><br>**Mother's name:** home address/cell phone number (length of residence, or other identifying information) may be written (not entered in DMS) | **2. Subject ID**<br>[Place label] | **3. Date of screening**<br>(dd-mm-yyyy) | **4. Is woman resident of cluster?**<br>1=Yes<br>2=No | **5. Village ID**<br>(residence at enrollment) | **6. Consent**<br>1=Yes<br>2=No<br>3=Ineligible | **7. Has mother been enrolled in the MNH before?**<br>1=Yes<br>2=No<br>3=DK | **8. MNH ID if known**<br>If Q7 =yes, write MNH ID.If MNH ID unknown, year of prior enrollment. *If missing ID and year than enter 99999* |
|---|---|---|---|---|---|---|---|
| | | | | \|__\|__\|__\|__\| | | | \|__\|__\|__\|__\|__\| |
| | | | | \|__\|__\|__\|__\| | | | \|__\|__\|__\|__\|__\| |
| | | | | \|__\|__\|__\|__\| | | | \|__\|__\|__\|__\|__\| |
| | | | | \|__\|__\|__\|__\| | | | \|__\|__\|__\|__\|__\| |
| | | | | \|__\|__\|__\|__\| | | | \|__\|__\|__\|__\|__\| |
