# Supplementary material for: Maternal and Newborn Health in Karnataka State, India: The Community Level Interventions for Pre-Eclampsia (CLIP) Trial’s Baseline Study Results
Source: PLoS One. 2017 Jan 20;12(1):e0166623. doi: 10.1371/journal.pone.0166623 (PMC5249209; doi:10.1371/journal.pone.0166623)
Supplement: S3 File — (PDF) [file pone.0166623.s003.pdf]

University of British Columbia
KLE University's JNMC & SNMC

Maternal Newborn Health Registry

ENROLLMENT FORM

MN01

SUBJECT ID:|___|___|___||___|___|___|___|___| |___| |___|

Version 2.0
July 16, 2013

This form should be completed by the Registry Administrator at the time the pregnant/delivered woman is screened and consented in the cluster (this may be any time during her pregnancy or after delivery).The goal is to enroll as early as possible; however, a woman may be enrolled at any point in pregnancy or after delivery.

A. MATERNAL INFORMATION

1. Did mother enroll prior to delivery? 1|__|Yes 2|__|No- **Skip to A3**

2. Planned delivery location:

1|__| Home→ village ID|__|__|__|__|__|__|
2|__| Facility→facility ID |__|__|__|__|__|__|
3|__| Other, specify ______________________________________
4|__| Don't know

3. Estimated delivery date: |__|__|-|__|__|-|__|__|__|__|
(enter 999 if unknown) dd mm yyyy

4. Method to obtain estimated delivery date (EDD) ***(Check all that apply)***

1|__| Date of last menstrual period: a.|__|__|-|__|__|-|__|__|__|__|
(enter 999 if unknown) dd mm yyyy
2|__| Clinical exam
3|__| USG b. |__|__|-|__|__|-|__|__|__|__|
(enter 999 if unknown) dd mm yyyy
4|__| Date unknown
5|__| Other →c.______________________________________

5. Maternal age at enrollment:|__|__| years (enter 99 if unknown)

6. Level of maternal schooling

1|__|No formal schooling, illiterate 2|__| No formal schooling, literate
3|__|Schooling → a. Years of schooling 1|__|__|
4|__|Don't know

7. Parity, excluding this pregnancy: |__|__|(enter 99 if unknown)

8. If you were pregnant (≥20 wks) before this pregnancy,

a. Did last pregnancy result in a live birth? 1|__|Yes 2|__|No
b. Date of last delivery: |__|__|-|__|__|-|__|__|__|__|
d d m m yyyy
*Enter 99 for parts or all of the date that are unknown**

9. Maternal height: |__|__|__| (enter 999 if unknown)

a. Unit of measurement: 1|__| cm 2|__| in
b. Method to determine height
1|__| Measured 2|__| Self Report 3|__| Not Available

10. Maternal weight: |__|__|__| (enter 999 if unknown)

a. Unit of measurement: 1|__| Kg 2|__| Lb
b. Indicate timing of weight
1|__|Pre-pregnancy or < 12 wks gestation 2|__|At visit
3|__|Other → c.______________________________
c. Method to determine weight
1|__| Measured 2|__| Self Report 3|__| Not Available

11. Hemoglobin: |__|__|.|__|gm/dl ☐ a. unavailable

B. FORM COMPLETION

1. Date form completed: |__|__|-|__|__|-|__|__|__|__|
d d m m yyyy
2. Name of person completing form:___________________________

a. ID: |__|__|__|__|

b. If applicable, Code of BA reporting birth: |__|__|__|__|
