# Supplementary material for: Maternal and Newborn Health in Karnataka State, India: The Community Level Interventions for Pre-Eclampsia (CLIP) Trial’s Baseline Study Results
Source: PLoS One. 2017 Jan 20;12(1):e0166623. doi: 10.1371/journal.pone.0166623 (PMC5249209; doi:10.1371/journal.pone.0166623)
Supplement: S4 File — (PDF) [file pone.0166623.s004.pdf]

ಕೆನಡಾದ ಬ್ರಿಟೀಷ್‌ಕೊಲಂಬಿಯಾ ವಿಶ್ವವಿದ್ಯಾಲಯ<br>ಕೆ. ಎಲ್. ಇ ವಿಶ್ವವಿದ್ಯಾಲಯದ<br>ಜೆ.ಎನ್.ಎಮ್.ಸಿ ಮತ್ತು ಎಸ್.ಎನ್.ಎಮ್.ಸಿ	**ತಾಯಿಯ ಹಾಗೂ ನವಜಾತ ಶಿಶುವಿನ ಆರೋಗ್ಯ ದಾಖಲಾತಿ<br>ದಾಖಲಾತಿ ನಮೂನೆ**	ಎಮ್.ಎನ್ 01
ಪುಟ 1 ರಲ್ಲಿ 1	ಭಾಗವಹಿಸುವವರ ಗುರುತು : |___|___|___|___|___|___|	ಆವತರಣೆ 2.0 ಜುಲೈ 16, 2013

ಈ ನಮೂನೆಯನ್ನು ಸಮೂದಾಯದಲ್ಲಿ ಗರ್ಭಿಣಿ / ಬಾಣಂತಿ ಸ್ತ್ರೀಯನ್ನು ತಪಾಸಣೆ ಮಾಡುವ ಹಾಗೂ ಒಪ್ಪಿಗೆ ಪಡೆಯುವ ಸಮಯದಲ್ಲಿ (ಇದು ಅವಳ ಗರ್ಭಾವಸ್ಥೆಯ ಯಾವುದೇ ಸಮಯದಲ್ಲಿ ಅಥವಾ ಹೆರಿಗೆ ನಂತರ ಇರಬಹುದು)ದಾಖಲಾತಿ ಆಡಳಿತಾಧಿಕಾರಿಯು ಪೂರ್ಣಗೊಳಿಸುವುದು. ಎಷ್ಟು ಸಾಧ್ಯವೋ ಅಷ್ಟು ಬೇಗನೆ ದಾಖಲಾತಿ ಮಾಡುವುದು ಗುರಿಯಾಗಿದೆ ಅದಾಗ್ಯೂ ಸ್ತ್ರೀಯನ್ನು ಗರ್ಭಾವಸ್ಥೆಯ ಯಾವುದೇ ಸಮಯದಲ್ಲಿ ಅಥವಾ ಹೆರಿಗೆ ನಂತರ ದಾಖಲಾತಿ ಮಾಡುಬಹುದು.

ಅ ತಾಯಿಯ ಮಾಹಿತಿ

1. ತಾಯಿಯು ಹೆರಿಗೆಯ ಮುಂಚೆ ದಾಖಲಾತಿಯನ್ನು ಮಾಡಿಸಿದ್ದಾಳೆಯೇ ? 1|__ ಹೌದು 2|__ ಇಲ್ಲ→ **ಪ್ರಶ್ನೆ ಅ3ಕ್ಕೆ ಹೋಗಿ**

2. ಯೋಜಿತ ಹೆರಿಗೆ ಸ್ಥಳ
   1|__ ಮನೆ→ಗ್ರಾಮ __________ ಗುರುತಿನ ಸಂಕೇತ |__|__|__|__|__|__|
   2|__ ಸೌಲಭ್ಯ→ಹೆಸರು__________ ಗುರುತಿನ ಸಂಕೇತ |__|__|__|__|__|__|
   3|__ ಇತರೆ, ನಮೂದಿಸಿ ____________________
   4|__ ಗೊತ್ತಿಲ್ಲ

3. ಅಂದಾಜಿಸಿದ ಹೆರಿಗೆಯ ದಿನಾಂಕ |__|__|-|__|__|-|__|__|__|__|
   (ಗೊತ್ತಿರದಿದ್ದರೆ 999 ಎಂದು ನಮೊದಿಸಿ) ದಿ ದಿ ತಿಂತಿಂ ವ ವ ವ ವ

4. ಹೆರಿಗೆಯ ದಿನಾಂಕವನ್ನು ನಿರ್ಧರಿಸುವ ವಿಧಾನ : **(ಅನ್ವಯಿಸುವುದೆಲ್ಲವನ್ನು ಗುರುತು ಹಾಕಿ.)**
   1|__ ಕೊನೆಯ ಮುಟ್ಟಿನ ದಿನಾಂಕ : ಅ. |__|__|-|__|__|-|__|__|__|__|
   (ಗೊತ್ತಿರದಿದ್ದರೆ 999 ಎಂದು ನಮೊದಿಸಿ) ದಿ ದಿ ತಿಂತಿಂ ವ ವ ವ ವ
   2|__ ವೈದ್ಯಕೀಯ ಪರೀಕ್ಷೆ
   3|__ ಅಲ್ಟ್ರಾಸೌಂಡ್ ಆ. |__|__|-|__|__|-|__|__|__|__|
   (ಗೊತ್ತಿರದಿದ್ದರೆ 999 ಎಂದು ನಮೂದಿಸಿ) ದಿ ದಿ ತಿಂತಿಂ ವ ವ ವ ವ
   4|__ ದಿನಾಂಕ ಗೊತ್ತಿಲ್ಲ
   5|__ ಇತರೆ→ ಇ.____________________

5. ದಾಖಲಾತಿ ಸಮಯದಲ್ಲಿ ತಾಯಿಯ ವಯಸ್ಸು: |__|__| ವರ್ಷಗಳು (ಗೊತ್ತಿರದಿದ್ದರೆ 99 ಎಂದು ನಮೂದಿಸಿ)

6. ತಾಯಿಯ ಶಾಲಾ ಮಟ್ಟ :
   1|__ ಔಪಚಾರಿಕ ಶಾಲಾಶಿಕ್ಷಣವಿಲ್ಲ, ಅನಕ್ಷರಸ್ಥಳು 2|__ಔಪಚಾರಿಕ ಶಾಲಾಶಿಕ್ಷಣವಿಲ್ಲ, ಅಕ್ಷರಸ್ಥಳು
   3|__ ಶಾಲಾಶಿಕ್ಷಣ→ಅ. ಪೂರ್ಣಗೊಳಿಸಿದ ವರ್ಷಗಳು |__|__|
   4|__ ಗೊತ್ತಿಲ್ಲ

7. ಹೆರಿಗೆಗಳ ಸಂಖ್ಯೆ(ಪ್ರಸಕ್ತ ಗರ್ಭಾವಸ್ಥೆಯನ್ನು ಹೊರತುಪಡಿಸಿ): |__|__| (ಗೊತ್ತಿರದಿದ್ದರೆ 99 ಎಂದು ನಮೂದಿಸಿ)

8. ಈ ಗರ್ಭಾವಸ್ಥೆಯ ಮೊದಲು, ನೀವು ಗರ್ಭಿಣಿಯಾಗಿದ್ದಲ್ಲಿ ($\geq$ 20 ವಾರಗಳು)
   ಅ. ಈ ಹಿಂದಿನ ಗರ್ಭಾವಸ್ಥೆಯ ಫಲಿತಾಂಶ ಸಜೀವ ಜನನವಾಗಿತ್ತೇ? 1|__ ಹೌದು 2|__ ಇಲ್ಲ
   ಆ. ಈ ಹಿಂದಿನ ಗರ್ಭಾವಸ್ಥೆಯ ಹೆರಿಗೆ ದಿನಾಂಕ: |__|__|-|__|__|-|__|__|__|__|
   ದಿ ದಿ ತಿಂತಿಂ ವ ವ ವ ವ

ಗೊತ್ತಿಲ್ಲದ ದಿನಾಂಕಗಳಿಗೆ ಅಥವಾ ಭಾಗಗಳಿಗೆ 99 ಎಂದು ನಮೂದಿಸಿ *

9. ತಾಯಿಯ ಎತ್ತರ: |__|__|__| (ಗೊತ್ತಿರದಿದ್ದರೆ 999 ಎಂದು ನಮೂದಿಸಿ)
   ಅ. ಅಳತೆಯ ಮಾಪ: 1|__ ಸೆಂ.ಮೀ. 2|__ ಇಂಚು
   ಬ. ಎತ್ತರ ನಿರ್ಧರಿಸಿದ ವಿಧಾನ
   1|__ ಅಳತೆ ಮಾಡಿದ್ದು 2|__ ಸ್ವಂತ ಹೇಳಿಕೆ 3|__ ಲಭ್ಯವಿಲ್ಲ

10. ತಾಯಿಯ ತೂಕ: |__|__|__| kg(ಗೊತ್ತಿರದಿದ್ದರೆ 999 ಎಂದು ನಮೂದಿಸಿ
   ಅ. ಅಳತೆಯ ಮಾಪ: 1|__ kg 2|__ Lb (ಪೌಂಡ)
   ಬ. ತೂಕ ಮಾಡಿದ ಅವಧಿ ಗುರುತಿಸಿ
   1|__ ಗರ್ಭಧಾರಣೆಯ ಪೂರ್ವದಲ್ಲಿ ಅಥವಾ <12 ವಾರಗಳ ಗರ್ಭಾವಸ್ಥೆಯಲ್ಲಿ
   2|__ ಭೇಟಿಯ ಸಮಯದಲ್ಲಿ
   3|__ ಇತರೆ → ನಮೂದಿಸಿ.____________________
   ಕ. ತೂಕ ನಿರ್ಧರಿಸಿದ ವಿಧಾನ
   1|__ ತೂಕ ಮಾಡಿದ್ದು 2|__ಸ್ವಂತ ಹೇಳಿಕೆ 3|__ ಲಭ್ಯವಿಲ್ಲ

11. ಹಿಮೋಗ್ಲೋಬಿನ್ : |__|__|. |__gm/dl ☐ಅ. ಲಭ್ಯವಿಲ್ಲ

ಬ. ನಮೂನೆಯನ್ನು ಪೂರ್ಣಗೊಳಿಸುವುದು

1. ನಮೂನೆಯನ್ನು ಪೂರ್ಣಗೊಳಿಸಿದ ದಿನಾಂಕ: |__|__|-|__|__|-|__|__|__|__|
   ದಿ ದಿ ತಿಂತಿಂ ವ ವ ವ ವ
2. ನಮೂನೆಯನ್ನು ಪೂರ್ಣಗೊಳಿಸಿದವರ ಹೆಸರು:____________________
   ಅ. ಗುರುತು: |__|__|__|__|
   ಬ.ಅನ್ವಯಿಸುವಂತಿದ್ದರೆ, ಜನನ ವರದಿಮಾಡುವವರ ಸಂಕೇತ : |__|__|__|__|
