# Supplementary material for: Maternal and Newborn Health in Karnataka State, India: The Community Level Interventions for Pre-Eclampsia (CLIP) Trial’s Baseline Study Results
Source: PLoS One. 2017 Jan 20;12(1):e0166623. doi: 10.1371/journal.pone.0166623 (PMC5249209; doi:10.1371/journal.pone.0166623)
Supplement: S5 File — (PDF) [file pone.0166623.s005.pdf]

University of British Columbia<br>KLE University's JNMC & SNMC	Maternal Newborn Health Registry<br>**SUPPLEMENTAL ENROLLMENT FORM**	MN01 (Supplemental)
**Page** 1	**SUBJECT ID: |___|___|___|___|___|___|**	**Version 6 2013/07/26**

This form should be completed by the Registry Administrator at the time the pregnant/delivered woman is screened and consented in the cluster (this may be any time during her pregnancy or after delivery). The goal is to enroll at <20 weeks; however, a woman may be enrolled at any point in pregnancy or after delivery.

A. DEMOGRAPHIC AND HOUSEHOLD INFORMATION

1. Religion
1|__| Hindu 2|__| Muslim 3|__| Christian 4|__|Sikh 5|__|Jain
6|__|Other

2. Level of husband's schooling
1|__| No formal schooling, illiterate 2|__| No formal schooling, literate
3|__| Schooling → a. Years of schooling 1|__|__| 4 |__| DK

3. BPL card holder 1|__| Yes 2|__| No 3|__| DK

4. Source of household drinking water
1|__| Faucet inside house (public water supply) 2|__| Public tap
3|__| Other pipe source 4|__| Public well 5|__| Mechanical pump well
6|__| River or canal 7|__| Borewell within the home
8|__| Other, specify ________________ 9|__| DK

5. Household sanitation
1|__| Own flush toilet 2|__| Shared flush toilet
3|__| Latrine/traditional pit toilet 4|__| Open air defecation
5|__| Other, specify___________ 6|__| DK

B. BIRTH PREPAREDNESS

1. Woman needs permission to seek emergency care
1|__| Yes 2|__| No 3|__| DK **(No/DK, skip to 3)**

2. Permission obtained to seek emergency care
1|__| Yes 2|__| No 3|__| DK

3. Has transportation plan for seeking emergency care
1|__| Yes 2|__| No 3|__| DK **(No/DK, skip to 6)**

4. Type of transport
1|__| Ambulance 2|__| Hired vehicle 3|__| Village vehicle
4|__| Own vehicle 5|__| Other, specify________________________

5. Estimated cost of transportation |__|__|__|__|__| rupees |__| DK

6. Emergency funds saved 1|__| Yes 2|__| No 3|__| DK

7. Community funds available for emergencies
1|__| Yes 2|__| No 3|__| DK

8. Contraception ever used 1|__| Yes 2|__| No 3|__| DK

9. Total number of pregnancies (including current pregnancy) |__|__|
If this is the first pregnancy, skip to Section F

C. CARE SEEKING IN LAST PREGNANCY

1. ANC care provider in **last** pregnancy
ANM 1|__| Yes 2|__| No 3|__| DK
Nurse 1|__| Yes 2|__| No 3|__| DK
Qualified doctor 1|__| Yes 2|__| No 3|__| DK

2. Number of times ANC sought from care provider in **last** pregnancy
ANM |__|__| Nurse |__|__| Qualified doctor |__|__|

3. Place of delivery in **last** pregnancy
1|__| Home (her/relative/friend/informal provider) 2|__| Sub-center
3|__| PHC 4|__| Government hospital 5|__| Private clinic/center
6|__| Private hospital 7|__| On route to formal health facility
8|__| Other 9|__| DK

D. MATERNAL OBSTETRIC INFORMATION

1. Outcome of previous pregnancies
a. Total number of miscarriages |__|__|
b. Total number of MTPs |__|__|
c. Total number of stillbirths |__|__|
d. Total number of live births |__|__|
e. Total number of early **[0-7 days]** neonatal deaths |__|__|
f. Total number of late **[8-28 days]** neonatal deaths |__|__|
g. Total number of living children |__|__|

This form should be completed by the Registry Administrator at the time the pregnant/delivered woman is screened and consented in the cluster (this may be any time during her pregnancy or after delivery). The goal is to enroll at <20 weeks; however, a woman may be enrolled at any point in pregnancy or after delivery.

E. PAST PREGNANCY

1. High blood pressure in any past pregnancy
1|__| Yes 2|__| No 3|__| DK

2. Eclampsia in any past pregnancy 1|__| Yes 2|__| No 3|__| DK

3. Diabetes mellitus in any past pregnancy
1|__| Yes 2|__| No 3|__| DK

F. MATERNAL MEDICAL INFORMATION
Pre-existing medical conditions

1. High blood pressure 1|__| Yes 2|__| No 3|__| DK

2. Epilepsy (fits) 1|__| Yes 2|__| No 3|__| DK

3. Diabetes mellitus 1|__| Yes 2|__| No 3|__| DK

4. TB [active or treated] 1|__| Yes 2|__| No 3|__| DK

5. History of worms (diagnosed or previously treated)
1|__| Yes 2|__| No 3|__| DK

6**.** Malaria medications taken in **<u>last</u>** year 1|__| Yes 2|__| No 3|__| DK

G. PRE-ECLAMPSIA KNOWLEDGE

1. **Aware** that women can have:

a. Abnormal bleeding after delivery 1|__| Yes 2|__| No 3|__| DK

b. Seizures during pregnancy 1|__| Yes 2|__| No 3|__| DK

c. High blood pressure in pregnancy 1|__| Yes 2|__| No 3|__| DK

d. High blood pressure in pregnancy that can be life-threatening
1|__| Yes 2|__| No 3|__| DK

2. Able to **spontaneously mention** the following warning symptoms of high blood pressure in pregnancy

The woman is asked to list warning symptoms that she is aware of. If she lists any of the symptoms below, check yes.

The woman should NOT be prompted.

1|__| Headache
2|__| Visual disturbance
3|__| Chest pain
4|__| Shortness of breath
5|__| Nausea and vomiting
6|__| Abdominal pain
7|__| Vaginal bleeding
8|__| Unconsciousness
9|__| Stroke
10|__| Seizures

H. FORM COMPLETION

1.Date form completed:
|__|__|-|__|__|-|__|__|__|__|
d d m m y y y y

2. Name of person completing form:______________________________
a. ID: |__|__|__|__|
b. If applicable, Code of BA reporting birth: |__|__|__|__|
