# Supplementary material for: Maternal and Newborn Health in Karnataka State, India: The Community Level Interventions for Pre-Eclampsia (CLIP) Trial’s Baseline Study Results
Source: PLoS One. 2017 Jan 20;12(1):e0166623. doi: 10.1371/journal.pone.0166623 (PMC5249209; doi:10.1371/journal.pone.0166623)
Supplement: S7 File — (PDF) [file pone.0166623.s007.pdf]

University of British Columbia<br>KLE University's JNMC & SNMC	Maternal Newborn Health Registry<br>**Supplemental PERINATAL FORM**	MN02 (Supplemental)
**Page** 1 of 3	**SUBJECT ID: |___|___|___|___|___|___|**	**Version 6 2013/07/26**

This form should be completed by the Registry Administrator for all deliveries reported and all maternal deaths.

A. MATERNAL PREVENTIVE TREATMENT during pregnancy

1.Took malaria medication 1|__| Yes 2|__| No 3|__|DK

2. Slept under mosquito net
1|__|Consistently 2|__| Occasionally 3|__| Never

3. Total number of meals and snacks per day
1|__| 1 2|__| 2 3|__| 3 4|__| 4 5|__| 5 6|__| 6 7|__| >6

B. MATERNAL COMMUNITY TREATMENT at any time

1. MgSO4 **injection** received
1|__| Yes 2|__| No 3|__| DK **(No/DK, skip to 4)**

2. Infection at injection site 1|__| Yes 2|__| No 3|__| DK

3. Haematoma at injection site 1|__| Yes 2|__| No 3|__| DK

4. CPR received 1|__| Yes 2|__| No 3|__| DK 4|__| N/A

C. CARE SEEKING other than for regular ANC or for delivery:

1. Care sought at PHC
1|__| Yes 2|__| No 3|__| DK **(No/DK, skip to 3)**

2. Number of PHC visits |__|__|

3. Care sought at facility higher than PHC
1|__| Yes 2|__| No 3|__| DK **(No/DK, skip to Section D)**

4. Number of visits to facility/facilities higher than PHC |__|__|

5. Admission to facility higher than PHC
1|__| Yes 2|__| No 3|__| DK **(No/DK, skip to Section D)**

6. Total number of overnight stays in facilities higher than PHC
|__|__|__| days

D. FACILITY PRESENTATION AND MATERNAL TREATMENT

1. For delivery or postpartum, care sought at a facility higher than PHC
1|__| Yes 2|__| No 3|__| DK **(If no/DK, skip to Section E)**

2. Date left home |__|__|-|__|__|-|__|__|__|__|
d d m m y y y y

3. Time left home |__|__| hours |__|__|minutes

4. Date of admission |__|__|-|__|__|-|__|__|__|__|
d d m m y y y y

5. Time of admission |__|__| hours |__|__|minutes

6. Last facility name ________________________ ID |__|__|__|__|

7. Mother discharged alive
1|__| Yes
2|__| No → ***Complete CLIP M VA/SA after completing this form***
3|__| NA/currently admitted **(Skip to 9)**

8. Date of maternal discharge/death |__|__|-|__|__| - __|__|__|__|
d d m m y y y y

9. Mother identified as having **any** health problem(s) on arrival at this facility or during her stay there
1|__| Yes 2|__| No 3|__| DK **(If no/DK, skip to Section E)**
(If yes, and the mother is currently still admitted, please finish completing this form after discharge.)

10. Arrived at this facility directly from which location
1|__| Community 2|__| Other facility 3|__| DK

11. Mother suffered transport-related injury
1|__| Yes 2|__| No 3|__| DK

12. Reasons for seeking care and reported problem(s) on arrival at final facility. (Please answer yes/no/DK for **each**. Check **all** that apply.)

	**12A. Reason for seeking care**			**12B. Present on arrival at final facility**		
	**Yes**	**No**	**DK**	**Yes**	**No**	**DK**
a. Labour pains	1|__|	2|__|	3|__|	1|__|	2|__|	3|__|
b. Prolonged labour	1|__|	2|__|	3|__|	1|__|	2|__|	3|__|
c. Ruptured membranes	1|__|	2|__|	3|__|	1|__|	2|__|	3|__|
d. Eclampsia warning symptoms **(No/DK, skip to e)**	1|__|	2|__|	3|__|	1|__|	2|__|	3|__|
i. Headache	1|__|	2|__|	3|__|	1|__|	2|__|	3|__|

This form should be completed by the Registry Administrator for all deliveries reported and all maternal deaths.

		Reason for seeking care				Present on arrival at final facility		
		Yes	No	DK		Yes	No	DK
ii. Visual disturbance		1|__|	2|__|	3|__|		1|__|	2|__|	3|__|
iii. Chest pain		1|__|	2|__|	3|__|		1|__|	2|__|	3|__|
iv. Shortness of breath		1|__|	2|__|	3|__|		1|__|	2|__|	3|__|
v. Abdominal pain		1|__|	2|__|	3|__|		1|__|	2|__|	3|__|
vi. Vaginal bleeding		1|__|	2|__|	3|__|		1|__|	2|__|	3|__|
e. Any other maternal health problems		1|__|	2|__|	3|__|		1|__|	2|__|	3|__|
f. Reduced or no fetal movements		1|__|	2|__|	3|__|		1|__|	2|__|	3|__|
g. Other		1|__|	2|__|	3|__|		1|__|	2|__|	3|__|

Signs on arrival at **this** facility

13. Systolic BP |__|__|__| mmHg 1|__| Not available

14. Diastolic BP |__|__|__| mmHg 1|__| Not available

15. HR |__|__|__| beats per minute 1|__| Not available

16. RR | __|__| respirations per minute 1|__| Not available

17. Oxygen saturation |__|__|__|% 1|__| Not available

18. Urine protein 1|__| None 2|__| Trace
3|__| 1+ 4|__| 2+ 5|__| 3+ 6|__| 4+ 7|__| DK/Not done

During her stay at **this** facility

19. sBP <110 mmHg at any time 1|__| Yes 2|__| No 3|__| DK

20. sBP ≥160 mmHg at any time 1|__| Yes 2|__| No 3|__| DK

21. Leukocyte count **highest** |__|__|__|__|__|x10^6/L 1|__| DK/Not done

22. Leukocyte count **lowest** |__|__|__|__|__|x10^6/L 1|__| DK/Not done

23. Received MgSO4
1|__| Yes 2|__| No 3|__| DK **(No/DK, skip to 27)**

24. Received MgSO4 by IM injection
1|__| Yes 2|__| No 3|__| DK **(No/DK, skip to 27)**

25. Infection at injection site 1|__| Yes 2|__| No 3|__| DK

26. Haematoma at injection site 1|__| Yes 2|__| No 3|__| DK

27. Received methyldopa 1|__| Yes 2|__| No 3|__| DK

28. Received other antihypertensive(s) 1|__| Yes 2|__| No 3|__| DK

29. Received any of the following life-saving interventions.
(Please answer yes/no/DK to **each**.)

	Yes	No	DK
a. CPR	1|__|	2|__|	3|__|
b. Mechanical ventilation	1|__|	2|__|	3|__|
c. Dialysis	1|__|	2|__|	3|__|
d. Anti-shock garment	1|__|	2|__|	3|__|
e. Bimanual uterine compression	1|__|	2|__|	3|__|
f. Brace sutures	1|__|	2|__|	3|__|
g. Internal iliac artery ligation/ devascularisation procedure	1|__|	2|__|	3|__|

E. MATERNAL OUTCOMES

1. Health problems suffered, either during pregnancy or postpartum. Please answer no/DK/yes to **each** condition. ***If 'yes' to any condition, specify where the health problem occurred***.

	No	DK	Yes →	Where outcome occurred		
				Community	On route after PHC	After arrival at last facility
a. Stroke	2|__|	3|__|	1|__|→	4|__|	5|__|	6|__|
b. Seizure (fits) **(No/DK skip to d)**	2|__|	3|__|	1|__|→	4|__|	5|__|	6|__|

This form should be completed by the Registry Administrator for all deliveries reported and all maternal deaths.

	No	DK	Yes →	Where outcome occurred		
				**Community**	**On route after PHC**	**After arrival at last facility**
c. Injury related to seizure	2|__|	3|__|	1|__|→	4|__|	5|__|	6|__|
d. Coma	2|__|	3|__|	1|__|→	4|__|	5|__|	6|__|
e. Fever (**No/ DK, skip to f**)	2|__|	3|__|	1|__|→	4|__|	5|__|	6|__|
i) Headache **and** stiff neck	2|__|	3|__|	1|__|→	4|__|	5|__|	6|__|
ii) Cough **and** shortness of breath	2|__|	3|__|	1|__|→	4|__|	5|__|	6|__|
iii) Abdominal pain/uterine tenderness	2|__|	3|__|	1|__|→	4|__|	5|__|	6|__|
iv) Painful urination or flank pain	2|__|	3|__|	1|__|→	4|__|	5|__|	6|__|
v) Foul-smelling vaginal discharge	2|__|	3|__|	1|__|→	4|__|	5|__|	6|__|
f. Antepartum haemorrhage	2|__|	3|__|	1|__|→	4|__|	5|__|	6|__|
g. Failure to form clots	2|__|	3|__|	1|__|→	4|__|	5|__|	6|__|

F. NEWBORN OUTCOMES

1. Multiple birth 1|__| Yes 2|__| No 3|__| DK

[If yes, complete MN02-Supplemental Perinatal Form (Sections F and G only) for twin using ID ending in 2, for triplets 3.]

2. Baby born alive 1|__| Yes 2|__| No **(If no, skip to Section G)**

3. Birth injury 1|__| Yes 2|__| No 3|__| DK

4. Newborn health problems after delivery.
(Please indicate yes/no/DK for **each**.)

	Yes	No	DK
a. Breathing problems	1|__|	2|__|	3|__|
b. Feeding problems	1|__|	2|__|	3|__|
c. Lethargy	1|__|	2|__|	3|__|
d. Coma	1|__|	2|__|	3|__|
e. Seizure (fits)	1|__|	2|__|	3|__|
f. Fever (>38° C)	1|__|	2|__|	3|__|
g. Umbilical stump infection	1|__|	2|__|	3|__|
h. Skin infection	1|__|	2|__|	3|__|
i. Jaundice	1|__|	2|__|	3|__|
j. Diarrhea/vomiting	1|__|	2|__|	3|__|
k. Bleeding	1|__|	2|__|	3|__|

5. Newborn admitted to facility for health problems
1|__| Yes 2|__| No 3|__| DK **(If no/DK, skip to Section G)**

6. Facility name ________________________ ID |__|__|__|__|

7. Date of admission |__|__|-|__|__| -|__|__|__|__|
d d m m y y y y

8. Newborn discharged alive
1|__| Yes
2|__| No → ***Complete CLIP P VA/SA after completing this form***
3|__| NA/currently admitted **(skip to Section G)**

9. Date of newborn discharge/death: |__|__|-|__|__| -|__|__|__|__|
d d m m y y y y

G. FORM COMPLETION

1. Date visit completed: |__|__|-|__|__|-|__|__|__|__|
d d m m y y y y

2. Name of person completing form: ______________________
a. ID: |__|__|__|__|
b. *If applicable,* Code of BA reporting birth: |__|__|__|__|

3. Location of data collection
1|__|Home 2|__|Health Center 3|__|Hospital
