# Supplementary material for: Maternal and Newborn Health in Karnataka State, India: The Community Level Interventions for Pre-Eclampsia (CLIP) Trial’s Baseline Study Results
Source: PLoS One. 2017 Jan 20;12(1):e0166623. doi: 10.1371/journal.pone.0166623 (PMC5249209; doi:10.1371/journal.pone.0166623)
Supplement: S9 File — (PDF) [file pone.0166623.s009.pdf]

ಕೆನಡಾದ ಬ್ರಿಟೀಷ್ ಕೊಲಂಬಿಯಾ ವಿಶ್ವವಿದ್ಯಾಲಯ<br>ಕೆ. ಎಲ್. ಇ ವಿಶ್ವವಿದ್ಯಾಲಯದ<br>ಜೆ.ಎನ್.ಎಮ್.ಸಿ ಮತ್ತು ಎಸ್.ಎನ್.ಎಮ್.ಸಿ	**ತಾಯಿಯ ಹಾಗೂ ನವಜಾತ ಶಿಶುವಿನ ಆರೋಗ್ಯ ದಾಖಲಾತಿ**<br>**ಅನುಸರಣೆಯ ನಮೂನೆ**	**ಎಮ್ಎನ್ 03**
**ಪುಟ1 ರಲ್ಲಿ1**	**ಭಾಗವಹಿಸುವವರ ಗುರುತು:**|___|___|___|___|___|___|___|___|___|___|	**ಆವತರಣೆ2.0**<br>**ಜೂಲೈ16, 2013**

ಈ ನಮೂನೆಯನ್ನುದಾಖಲಾತಿ ಆಡಳಿತಾಧಿಕಾರಿಯು ಪೂರ್ಣಗೊಳಿಸಬೇಕು. ಹೆರಿಗೆಯಾದ 6 ವಾರಗಳ ನಂತರ (ಶಿಶು ಅಥವಾ ತಾಯಿ 6 ವಾರಗಳೊಳಗೆ ಮರಣ ಹೊಂದಿದ್ದರೂ ಕೂಡ) ತಾಯಿ ಹಾಗೂ ಮಗುವಿನ ಸ್ಥಿತಿಯನ್ನು ನಿರ್ಣಯಿಸುವ ಉದ್ದೇಶಕ್ಕಾಗಿ ನಮೂನೆಯನ್ನು ಹೆರಿಗೆಯಾದ ಕನಿಷ್ಠ 6 ವಾರಗಳ ನಂತರ ಪೂರ್ಣಗೊಳಿಸಬೇಕು (ತಾಯಿ / ಶಿಶು ಚಿಕಿತ್ಸಾಲಯಕ್ಕೆ ಬೆಟ್ಟಿಕೊಡದಿದ್ದರೆ ಸ್ಥಿತಿಯನ್ನು ನಿರ್ಣಯಿಸಲು ಅವರ ಮನೆಯಲ್ಲಿ ಭೇಟಿ ಮಾಡಬೇಕು).ಮಹಿಳೆ ಹೆರಿಗೆ ಪೂರ್ವಮರಣ ಹೊಂದಿದ್ದರೆ ಅಥವಾ ಎಮ್.ಎನ್ 02 ನಮೂನೆಯಲ್ಲಿ ತಾಯಿ ಮತ್ತು ಶಿಶುವಿನ ಸ್ಥಿತಿ ಮರಣ ಎಂದು ನಮೂದಿಸಿದ್ದರೆ ಈ ನಮೂನೆಯನ್ನುಪೂರ್ಣಗೊಳಿಸುವ ಅವಶ್ಯ ಇರುವುದಿಲ್ಲ.

ಅ. ಶಿಶುವಿನ ಸ್ಥಿತಿ (ಎಮ್.ಎನ್ 02 ನಮೂನೆಯಲ್ಲಿ ಶಿಶುಮರಣ / ನಿರ್ಜೀವ ಜನನ / ಗರ್ಭಪಾತ ಎಂದು ನಮೂದಿಸಿದ್ದರೆ 'ಆ' ವಿಭಾಗಕ್ಕೆ ಹೋಗಿ)

1. ಶಿಶುವಿನ ಸ್ಥಿತಿ :
   - 1__ ಜೀವಂತವಾಗಿದೆ
   - 2__ ಮರಣ ಹೊಂದಿದೆ→ಮರಣದ ಕಾರಣಕ್ಕಾಗಿ ಪೆರಿನಾಟಲ್ (ಎಮ್ಎನ್05) ನಮೂನೆಯನ್ನು ತುಂಬಿರಿ
   - 3__ ಗೊತ್ತಿಲ್ಲ (ಮಗುವಿನ ಸ್ಥಿತಿಯನ್ನು ಪತ್ತೆ ಮಾಡಲು ಎಲ್ಲಾ ಪ್ರಯತ್ನಗಳನ್ನು ಮಾಡಿ)

2. ಪ್ರಸವದ ನಂತರ ಶಿಶುವಿಗೇನಾದರೂ ಪ್ರತಿಕೂಲ ಪರಿಸ್ಥಿತಿ ಇತ್ತೇ? (ಪ್ರತಿಯೊಂದಕ್ಕೂ ಗುರುತು ಹಾಕಿ)

	**ಹೌದು**	**ಇಲ್ಲ**	**ಗೊತ್ತಿಲ್ಲ**
ಅ. ಹುಟ್ಟು ನ್ಯೂನ್ಯತೆಗಳು (ಕಂಜೆನೈಟಲ್ ಅನಾಮಲಿ)	1__	2__	3__
ಅ1.ಹೌದಾದರೆ, ನಮೂದಿಸಿ:____________________			
ಆ. ತೀವ್ರಕಾಮಾಲೆ (ಹಸ್ತ / ಪಾದ)	1__	2__	3__
ಇ. ಮಗುವಿಗೇನಾದರು ಗಂಭೀರವಾದ ಸೋಂಕು (ಹೊಕ್ಕುಳ ಬಳ್ಳಿಯಲ್ಲಿ ಸೋಂಕು, ನಿಮೊನಿಯಾ , ಸೆಪ್ಸಿಸ್)→	1__	2__	3__

ಹೌದಾದರೆ, ನಮೂದಿಸಿ ಇ1.

____

ಬ. ತಾಯಿಯ ಸ್ಥಿತಿ

1. ಭೇಟಿ ಕೊಟ್ಟಾಗ ತಾಯಿಯ ಸ್ಥಿತಿ :
   - 1__ ಜೀವಂತವಾಗಿದ್ದಾಳೆ
   - 2__ಮರಣ ಹೊಂದಿದ್ದಾಳೆ→ಮರಣದ ಕಾರಣಕ್ಕಾಗಿ ಮೆಟರ್‌ನಲ್ ವರ್ಬಲ್ ಮತ್ತು ಸೋಶಿಯಲ್ ಅಟಾಪ್ಸಿ ನಮೂನೆಯನ್ನುತುಂಬಿ
   - 3__ಗೊತ್ತಿಲ್ಲ (ತಾಯಿಯ ಸ್ಥಿತಿಯನ್ನು ಪತ್ತೆ ಮಾಡಲು ಎಲ್ಲಾ ಪ್ರಯತ್ನಗಳನ್ನು ಮಾಡಿ)

	**ಹೌದು**	**ಇಲ್ಲ**	**ಗೊತ್ತಿಲ್ಲ**
2. ತಾಯಿಗೇನಾದರೂ ಅತೀಯಾದ ರಕ್ತಹೀನತೆ ಇರುತ್ತದೆಯೇ?	1__	2__	3__
3. ತಾಯಿಗೇನಾದರೂ ಪ್ರಸೂತಿದ್ವಾರದ ನಾಳವ್ರಣದ(ಫಿಸ್ಟುಲಾ) ಚಿಹ್ನೆ /ಚಿಹ್ನೆಗಳು/ಲಕ್ಷಣಗಳು?	1__	2__	3__

ಕ. ನಮೂನೆಯನ್ನು ಪೂರ್ಣಗೊಳಿಸುವುದು

1. ನಮೂನೆಯನ್ನು ಪೂರ್ಣಗೊಳಿಸಿದ ದಿನಾಂಕ: \|_\|_\| \|_\|_\| \|_\|_\|_\|_\|
   ದಿ ದಿ ತಿಂ ತಿಂ ವ ವ ವ ವ

2. ನಮೂನೆಯನ್ನು ಪೂರ್ಣಗೊಳಿಸಿದವರ ಹೆಸರು:__________________________
   ಅ. ಗುರುತು : \|_\|_\|_\|_\|
   ಬ.ಅನ್ವಯಿಸುವಂತಿದ್ದರೆ, ಜನನ ವರದಿ ಮಾಡುವವರ ಸಂಕೇತ : \|_\|_\|_\|_\|

3. ನಮೂನೆ ಪೂರ್ಣಗೊಳಿಸಿದ ಸ್ಥಳ : 1__ ಮನೆ 2__ ಆರೋಗ್ಯಕೇಂದ್ರ 3__ ಆಸ್ಪತ್ರೆ
