# Supplementary material for: Maternal and Newborn Health in Karnataka State, India: The Community Level Interventions for Pre-Eclampsia (CLIP) Trial’s Baseline Study Results
Source: PLoS One. 2017 Jan 20;12(1):e0166623. doi: 10.1371/journal.pone.0166623 (PMC5249209; doi:10.1371/journal.pone.0166623)
Supplement: S6 File — (PDF) [file pone.0166623.s006.pdf]

University of British Columbia<br>KLE University's JNMC & SNMC	Maternal Newborn Health Registry<br><br>PERINATAL FORM	MN02
	**SUBJECT ID:|___|___|___|___|___|___|___|___| ___|___|**	**Version 2.0<br>July 16, 2013**

This form should be completed by the Registry Administrator for all deliveries and all maternal deaths.

A. STATUS of MOTHER and HEALTH CARE SERVICES

1. Did mother have any antenatal care? 1|__|Yes 2|__|No
   3|__|Don't Know (DK)
   a. If yes, how many antenatal visits did she have?1|__|__|
   b. If yes, estimated date of mother's ***first*** antenatal care visit?
   |__|__|-|__|__|-|__|__|__|__|
   d d m m yyyy (enter 999 if unknown)

2. Did mother receive any of the following *during* ***this*** *pregnancy:*

		Yes	No	DK
a.	Tetanus toxoid vaccine	1|__|	2|__|	3|__|
b.	Iron	1|__|	2|__|	3|__|
c.	Vitamins, calcium	1|__|	2|__|	3|__|
d.	HIV test	1|__|	2|__|	3|__|
e.	Blood pressure measurement	1|__|	2|__|	3|__|
f.	Urine test for protein	1|__|	2|__|	3|__|
g.	Ultrasound for gestational age/other	1|__|	2|__|	3|__|

3. Maternal status at visit: 1|__| Alive
   2|__| Died→***If died, complete MN06 Maternal Mortality Form***

4. Date of delivery: |__|__|-|__|__|-|__|__|__|__|
   dd mm yyyy

5. Who conducted delivery? (Check one, highest level of provider)
   1|__| Obstetrician 2|__| Non-OB Physician
   3|__| Nurse/nurse midwife 4|__| Traditional Birth Attendant
   5|__| Family (no health provider) 6|__| Self delivery
   7|__| Other a. ___________________ 8|__| Don't Know

6. Where did delivery occur?
   1|__| Hospital
   2|__| Clinic/health center → a. Facility Name _______________ b. Facility ID___________________
   3|__| Home in Village→ c. Village Name ________________ d. Village ID _______________
   4|__| Other →e.____

7. Mode of delivery
   1|__| Vaginal (with forceps/vacuum)
   2|__| Vaginal (**without** forceps/vacuum)
   3|__| C-section
   4|__| Miscarriage
   5|__| Medical termination of pregnancy (MTP)

8. Did the mother have any of the following conditions?
   (*during* ***this*** *pregnancy*)

		Yes	No	DK
a.	Obstructed/prolonged labor/failure to progress	1|__|	2|__|	3|__|
b.	Major antepartum hemorrhage	1|__|	2|__|	3|__|
c.	Major postpartum hemorrhage	1|__|	2|__|	3|__|
d.	Evidence of hypertensive disease/severe pre-eclampsia/eclampsia	1|__|	2|__|	3|__|
e.	Breech/transverse or oblique lie	1|__|	2|__|	3|__|

9. Did the mother experience **any symptoms or signs of life threatening illness** at any time during the pregnancy, or at delivery?
   1|__| Yes **– *COMPLETE MN07*** 2|__| No 3|__| Don't Know

B. MATERNAL TREATMENT (Complete for all women)

Maternal treatment provided	Yes	No	DK
1. Antibiotics	1|__|	2|__|	3|__|
2. Corticosteroids	1|__|	2|__|	3|__|
3. Oxytocin or misoprostol	1|__|	2|__|	3|__|
4. Blood transfusion	1|__|	2|__|	3|__|
5. D&C or suction	1|__|	2|__|	3|__|
6. Magnesium sulfate	1|__|	2|__|	3|__|
7. Hysterectomy	1|__|	2|__|	3|__|
8. Episiotomy	1|__|	2|__|	3|__|

University of British Columbia<br>KLE University's JNMC & SNMC	Maternal Newborn Health Registry<br><br>PERINATAL FORM	MN02
**Page** 2 of 2	**SUBJECT ID:**|___|___|___|___|___|___|___|___| ___|___|	**Version 2.0**<br>**July 16, 2013**

This form should be completed by the Registry Administrator for all deliveries and all maternal deaths.

C. NEONATAL CONDITIONS AND OUTCOME

1. Fetal/Neonatal outcome

1|__|Miscarriage(<20wks)→**Skip to E1**

2|__|Medically terminated pregnancy (MTP) →**Skip to E1**

3|__|Stillbirth→ ***no movement, heartbeat or gasping***

4|__|Born alive, died before visit

5|__|Born alive, alive at visit

If stillborn or newborn died, complete MN05 cause of death form

2. Sex of the baby: 1|__| Male 2|__| Female 3|__| DK

3. Birth weight: |__|__|__|__| grams (if unknown enter '9999')

4. Date baby weighed: |__|__|-|__|__|-|__|__|__|__|
dd mm yyyy

5. If birth weight unknown, baby appeared to be:

1|__| Very small (<1000g) 2|__| Small (1000–1499g)

3|__| Small to normal (1500–2499g) 4|__| Normal $\geq$2500g

5|__| Don't Know

6. Was this a multiple birth? 1|__| Yes 2|__| No

If Yes, complete MN02, MN03 data forms for twin using ID ending in 2, for triplets 3

7. Was baby resuscitated?

1|__| Yes 2|__| No →**Skip to C9** 3|__| DK →**Skip to C9**

8. If baby was resuscitated, was resuscitation with bag and mask?

1|__| Yes 2|__| No 3|__| DK

9. Was baby placed on mother's chest after delivery?

1|__| Yes 2|__| No 3|__| DK 4|__| NA

10. Was baby bathed within 6 hours after delivery?

1|__| Yes 2|__| No 3|__| DK 4|__| NA

11. Did baby breastfeed within 1 hour after delivery?

1|__| Yes 2|__| No 3|__| DK 4|__| NA

D. NEONATAL TREATMENT (Complete for all infants)

Neonatal treatment provided.	Yes	No	DK
1. Antibiotics	1|__|	2|__|	3|__|
2. CPAP	1|__|	2|__|	3|__|
3. Oxygen	1|__|	2|__|	3|__|
4. Ventilation	1|__|	2|__|	3|__|
5. Medicinal eye care	1|__|	2|__|	3|__|
6. Cord care	1|__|	2|__|	3|__|

E. FORM COMPLETION

1. Date visit completed: |__|__|-|__|__|-|__|__|__|__|
dd mm yyyy

2. Name of person completing form: ____________________

a. ID: |__|__|__|__|

b. *If applicable,* Code of BA reporting birth: |__|__|__|__|

3. Location of data collection

1|__|Home 2|__|Health Center 3|__|Hospital
