# Supplementary material for: Maternal and Newborn Health in Karnataka State, India: The Community Level Interventions for Pre-Eclampsia (CLIP) Trial’s Baseline Study Results
Source: PLoS One. 2017 Jan 20;12(1):e0166623. doi: 10.1371/journal.pone.0166623 (PMC5249209; doi:10.1371/journal.pone.0166623)
Supplement: S8 File — (PDF) [file pone.0166623.s008.pdf]

**University of British Columbia**<br>**KLE University's JNMC & SNMC**	**Maternal Newborn Health Registry**<br><br>**FOLLOW-UP FORM**	**MN03**
**Page** 1 of 1	**SUBJECT ID:|___|___|___|___|___|___|___|___|___|___|**	**Version 2.0**<br>**July 16, 2013**

This form should be completed by the Registry Administrator. The purpose is to assess the status of the mother and baby 6 weeks after delivery (even if the baby or mother died before 6 weeks). The form should be completed at least 6 weeks after delivery (if mother/baby do not attend a clinic visit, a home visit should be made to determine status). *This form need not be completed if the woman died before delivery or if the status of both mother and newborn is dead on MN02.*

A. INFANT STATUS (Skip to Section B if baby noted as dead/ stillbirth/miscarriage on MN02)

1. Status of infant: 1|__| Alive
   2|__| Died → **Complete MN05** cause of death form
   3|__| Unknown (try to identify baby's status)

2. Did baby have any adverse conditions after delivery? (Check for each)

	Yes	No	DK
a. Congenital anomalies (birth defect)<br>a1. If yes, specify:______________________	1|__|	2|__|	3|__|
b. Severe jaundice (palms/soles)	1|__|	2|__|	3|__|
c. Did baby have a severe infection (omphalitis, pneumonia, sepsis)→<br>If yes, specify c1.<br>______________________________	1|__|	2|__|	3|__|

B. MATERNAL STATUS

1. Status of mother at visit:
   1|__| Alive
   2|__| Died → **Complete MN06** cause of death form
   3|__| Unknown (try to locate mother's status)

	Yes	No	DK
2. Did mother have severe anemia?	1|__|	2|__|	3|__|
3. Did mother have signs of obstetric fistula	1|__|	2|__|	3|__|

C. FORM COMPLETION

1. Date visit completed: |__|__|-|__|__|-|__|__|__|__|
   d d m m y yyy
2. Person completing form:______________________________
   a. ID: |__|__|__|__|
   b. If applicable, Code of BA reporting birth: |__|__|__|__|
3. Location of data collection: 1|__|Home 2|__|Health Center 3|__|Hospital
