# Supplementary material for: Maternal and Newborn Health in Karnataka State, India: The Community Level Interventions for Pre-Eclampsia (CLIP) Trial’s Baseline Study Results
Source: PLoS One. 2017 Jan 20;12(1):e0166623. doi: 10.1371/journal.pone.0166623 (PMC5249209; doi:10.1371/journal.pone.0166623)
Supplement: S10 File — (PDF) [file pone.0166623.s010.pdf]

University of British Columbia<br>KLE University's JNMC & SNMC	Maternal Newborn Health Registry<br>**SUPPLEMENTAL - FOLLOW-UP FORM**	MN03 (Supplemental)
	**SUBJECT ID: |___|___|___|___|___|___|**	**Version 6 2013/07/26**

This form covers events that may have occurred since completion of the MN02 Perinatal Form (and MN02 Supplemental Perinatal Form).
If the mother was not alive at the time of MN02, complete only Sections F and G.

A. COMMUNITY TREATMENT (Maternal)

1. MgSO4 **injection** received
1|__| Yes 2|__| No 3|__| DK **(No/DK, skip to 4)**

2. Infection at injection site 1|__| Yes 2|__| No 3|__| DK

3. Haematoma at injection site 1|__| Yes 2|__| No 3|__| DK

4. CPR received 1|__| Yes 2|__| No 3|__| DK 4|__| NA

B. CARE SEEKING for other than routine postnatal care

1. Care sought at PHC
1|__| Yes 2|__| No 3|__| DK **(No/DK, skip to 3)**

2. Number of PHC visits |__|__|

3. Care sought at facility higher than PHC
1|__| Yes 2|__| No 3|__| DK **(No/DK, skip to Section C)**

4. Number of visits to facility/facilities higher than PHC |__|__|

5. Admission to facility higher than PHC
1|__| Yes 2|__| No 3|__| DK **(No/DK, skip to Section C)**

6. Total N overnight stays in facilities higher than PHC |__|__|__| days

C. FACILITY PRESENTATION AND TREATMENT (Maternal)

1. Mother delivered in facility higher than PHC
1|__| Yes 2|__| No **(If no, skip to 4)**

2. Mother still in facility at the time of MN02
1|__| Yes 2|__| No 3|__| DK **(If no/DK, skip to 4)**

3. Date of maternal discharge/death |__|__|-|__|__|-|__|__|__|__|
d d m m y y y y

If DK/not yet discharged, Number of days stayed in facility |__|__| d

4. Mother identified as having **any** new health problem that developed since completion of MN02
1|__| Yes 2|__| No 3|__| DK **(If no/DK, skip to Section E)**

5. Mother delivered in the community
1|__| Yes 2|__| No **(If yes, skip to Section C.2)**

6. When did the new health problem(s) first develop?
(Check **all** that apply)
1|__| Before discharge for delivery admission → **Complete Section C.1**
2|__| After discharge for delivery admission or if no delivery admission occurred → **Complete Section C.2**

Section C.1

1. Last facility where mother admitted for this problem
Facility name ________________________ ID |__|__|__|__|

2. Mother discharged alive
1|__| Yes
2|__| No → ***Complete CLIP M VA/SA after completing this form***
3|__| NA/currently admitted **(Skip to 4)**

3. Date of maternal discharge/death |__|__|-|__|__|-|__|__|__|__|
d d m m y y y y

If DK, number of days stayed in facility |__|__| d

4. Arrived at this facility from which location
1|__| Community 2|__| Other facility 3|__| DK

5. Mother suffered transport-related injury
1|__| Yes 2|__| No 3|__| DK

6. Reasons for seeking care and reported problem(s) on arrival at **final** facility. (Please answer yes/no/DK for **each**.)

	6A. Reason for seeking care			6B. Present on arrival at facility		
	**Yes**	**No**	**DK**	**Yes**	**No**	**DK**
a. Labour pains	1|__|	2|__|	3|__|	1|__|	2|__|	3|__|
b. Prolonged labour	1|__|	2|__|	3|__|	1|__|	2|__|	3|__|
c. Ruptured membranes	1|__|	2|__|	3|__|	1|__|	2|__|	3|__|
d. Eclampsia warning symptoms **(No/DK, skip to e)**	1|__|	2|__|	3|__|	1|__|	2|__|	3|__|
i. Headache	1|__|	2|__|	3|__|	1|__|	2|__|	3|__|
ii. Visual	1|__|	2|__|	3|__|	1|__|	2|__|	3|__|

This form covers events that may have occurred since completion of the MN02 Perinatal Form (and MN02 Supplemental Perinatal Form).

If the mother was not alive at the time of MN02, complete only Sections F and G.

disturbance						
	**6A. Reason for seeking care**			**6B. Present on arrival at final facility**		
	**Yes**	**No**	**DK**	**Yes**	**No**	**DK**
iii. Chest pain	1|__|	2|__|	3|__|	1|__|	2|__|	3|__|
iv. Shortness of breath	1|__|	2|__|	3|__|	1|__|	2|__|	3|__|
v. Abdominal pain	1|__|	2|__|	3|__|	1|__|	2|__|	3|__|
vi. Vaginal bleeding	1|__|	2|__|	3|__|	1|__|	2|__|	3|__|
e. Any other maternal health problems	1|__|	2|__|	3|__|	1|__|	2|__|	3|__|
f. Reduced or no fetal movements	1|__|	2|__|	3|__|	1|__|	2|__|	3|__|
g. Other	1|__|	2|__|	3|__|	1|__|	2|__|	3|__|

<u>Signs on arrival at **this** facility</u>

7. Systolic BP |__|__|__| mmHg (999) 1|__| Not available

8. Diastolic BP |__|__|__| mmHg (999) 1|__| Not available

9. HR |__|__|__| beats per minute 1|__| Not available

10. RR |__|__| respirations per minute 1|__| Not available

11. Oxygen saturation |__|__|__|% 1|__| Not available

12. Urine protein 1|__| None 2|__| Trace
3|__| 1+ 4|__| 2+ 5|__| 3+ 6|__| 4+ 7|__| DK/ Not done

<u>During her stay at **this** facility</u>

13. sBP <110 mmHg at any time 1|__| Yes 2|__| No 3|__| DK

14. sBP ≥160 mmHg at any time 1|__| Yes 2|__| No 3|__| DK

15. Leukocyte count **highest** |__|__|__|__|__|x10^6/L 1|__| DK/Not done

16. Leukocyte count **lowest** |__|__|__|__|__| x10^6/L 1|__| DK/Not done

17. Received MgSO4
1|__| Yes 2|__| No 3|__| DK **(No/DK, skip to 21)**

18. Received MgSO4 by IM injection
1|__| Yes 2|__| No 3|__| DK **(No/DK, skip to 21)**

19. Infection at injection site 1|__| Yes 2|__| No 3|__| DK

20. Haematoma at injection site 1|__| Yes 2|__| No 3|__| DK

21. Received methyldopa 1|__| Yes 2|__| No 3|__| DK

22. Received other antihypertensive(s) 1|__| Yes 2|__| No 3|__| DK

23. Received any of the following life-saving interventions.
(Please answer yes/no/DK to <u>**each**</u>.)

	**Yes**	**No**	**DK**
a. CPR	1|__|	2|__|	3|__|
b. Mechanical ventilation	1|__|	2|__|	3|__|
c. Dialysis	1|__|	2|__|	3|__|
d. Anti-shock garment	1|__|	2|__|	3|__|
e. Bimanual uterine compression	1|__|	2|__|	3|__|
f. Brace sutures	1|__|	2|__|	3|__|
g. Internal iliac artery ligation/ devascularisation procedure	1|__|	2|__|	3|__|

Complete Section C.2 if another health problem developed <u>after</u> discharge for the delivery admission. If not, skip to Section D.

<u>Section C.2</u>

1. Mother admitted to a facility higher than PHC for this problem?
1|__| Yes 2|__| No 3|__| DK **(If no/DK, skip to Section D)**

2. Date left community |__|__|-|__|__|-|__|__|__|__|
d d m m y y y y

3. Time left community |__|__| hours |__|__|minutes

4. Date of admission |__|__|-|__|__|-|__|__|__|__|
d d m m y y y y

5. Time of admission |__|__| hours |__|__|minutes

6. Last facility name ________________________ ID |__|__|__|__|

University of British Columbia<br>KLE University's JNMC & SNMC	Maternal Newborn Health Registry<br>**SUPPLEMENTAL - FOLLOW-UP FORM**	**MN03 (Supplemental)**
	**SUBJECT ID: |___|___|___|___|___|___|**	**Version 6 2013/07/26**

This form covers events that may have occurred since completion of the MN02 Perinatal Form (and MN02 Supplemental Perinatal Form).
If the mother was not alive at the time of MN02, complete only Sections F and G.

7. Mother discharged alive
1|__| Yes
2|__| No → ***Complete CLIP M VA/SA after completing this form***
3|__| NA/currently admitted (**Skip to 9**)

8. Date of maternal discharge/death |__|__|-|__|__| - __|__|__|__| (d d, m m, y y y y)

If DK, Number of days stayed in facility |__|__| d

9. Arrived at this facility directly from which location
1|__| Community 2|__| Other facility 3|__| DK

10. Mother suffered transport-related injury
1|__| Yes 2|__| No 3|__| DK

11. Reasons for seeking care and reported problem(s) on arrival at final facility. (Please answer yes/no/DK for **each**. Check **all** that apply.)

	11A. Reason for seeking care			11B. Present on arrival at final facility		
	**Yes**	**No**	**DK**	**Yes**	**No**	**DK**
a. Eclampsia warning symptoms (**No/DK, skip to b**)	1|__|	2|__|	3|__|	1|__|	2|__|	3|__|
i. Headache	1|__|	2|__|	3|__|	1|__|	2|__|	3|__|
ii. Visual disturbance	1|__|	2|__|	3|__|	1|__|	2|__|	3|__|
iii. Chest pain	1|__|	2|__|	3|__|	1|__|	2|__|	3|__|
iv. Shortness of breath	1|__|	2|__|	3|__|	1|__|	2|__|	3|__|
v. Abdominal pain	1|__|	2|__|	3|__|	1|__|	2|__|	3|__|
vi. Vaginal bleeding	1|__|	2|__|	3|__|	1|__|	2|__|	3|__|
b. Any other maternal health problems	1|__|	2|__|	3|__|	1|__|	2|__|	3|__|
c. Other	1|__|	2|__|	3|__|	1|__|	2|__|	3|__|

Signs on arrival at **this** facility

12. Systolic BP |__|__|__| mmHg 1|__| Not available
13. Diastolic BP |__|__|__| mmHg 1|__| Not available
14. HR |__|__|__| beats per minute 1|__| Not available
15. RR |__|__| respirations per minute 1|__| Not available
16. Oxygen saturation |__|__|__|% 1|__| Not available
17. Urine protein 1|__| None 2|__| Trace
3|__| 1+ 4|__| 2+ 5|__| 3+ 6|__| 4+ 7|__| DK/Not done

During her stay at **this** facility

18. sBP <110 mmHg at any time 1|__| Yes 2|__| No 3|__| DK
19. sBP ≥160 mmHg at any time 1|__| Yes 2|__| No 3|__| DK
20. Leukocyte count **highest** |__|__|__|__|__|x10^6/L 1|__| DK/Not done
21. Leukocyte count **lowest** |__|__|__|__|__| x10^6/L 1|__| DK/Not done
22. Received MgSO4
1|__| Yes 2|__| No 3|__| DK (**No/DK, skip to 26**)
23. Received MgSO4 by IM injection
1|__| Yes 2|__| No 3|__| DK (**No/DK, skip to 26**)
24. Infection at injection site 1|__| Yes 2|__| No 3|__| DK
25. Haematoma at injection site 1|__| Yes 2|__| No 3|__| DK
26. Received methyldopa 1|__| Yes 2|__| No 3|__| DK
27. Received other antihypertensive(s) 1|__| Yes 2|__| No 3|__| DK
28. Received any of the following life-saving interventions. Please answer 'yes', 'no', or 'DK' to **each**.

	**Yes**	**No**	**DK**
a. CPR	1|__|	2|__|	3|__|
b. Mechanical ventilation	1|__|	2|__|	3|__|

This form covers events that may have occurred since completion of the MN02 Perinatal Form (and MN02 Supplemental Perinatal Form).
If the mother was not alive at the time of MN02, complete only Sections F and G.

c. Dialysis	1|__|	2|__|	3|__|
d. Anti-shock garment	1|__|	2|__|	3|__|
	**Yes**	**No**	**DK**
e. Bimanual uterine compression	1|__|	2|__|	3|__|
f. Brace sutures	1|__|	2|__|	3|__|
g. Internal iliac artery ligation/ devascularisation procedure	1|__|	2|__|	3|__|

D. MATERNAL OUTCOMES

1. Health problems suffered postpartum since completion of MN02. Please answer no/DK/yes to **each** condition. ***If yes to any condition, specify where the health problem occurred***.

	No	DK	Yes →	Where did this outcome occur?		
				**Community**	**On route after PHC**	**After arrival at last facility**
a. Stroke	2|__|	3|__|	1|__|→	4|__|	5|__|	6|__|
b. Seizure (fits) **(No/DK, skip to d)**	2|__|	3|__|	1|__|→	4|__|	5|__|	6|__|
c. Injury related to seizure	2|__|	3|__|	1|__|→	4|__|	5|__|	6|__|
d. Coma	2|__|	3|__|	1|__|→	4|__|	5|__|	6|__|
e. Fever **(No/DK, skip to f)**	2|__|	3|__|	1|__|→	4|__|	5|__|	6|__|
i. Headache **and** stiff neck	2|__|	3|__|	1|__|→	4|__|	5|__|	6|__|
ii Cough **and** shortness of breath	2|__|	3|__|	1|__|→	4|__|	5|__|	6|__|
iii. Abdominal pain/uterine tenderness	2|__|	3|__|	1|__|→	4|__|	5|__|	6|__|
iv. Painful urination or flank pain	2|__|	3|__|	1|__|→	4|__|	5|__|	6|__|
	**No**	**DK**	**Yes →**	**Where did this outcome occur?**		
				**Community**	**On route after PHC**	**After arrival at last facility**
v. Foul smell-ing vaginal discharge	2|__|	3|__|	1|__|→	4|__|	5|__|	6|__|
f. Failure to form clots	2|__|	3|__|	1|__|→	4|__|	5|__|	6|__|

E. MATERNAL FUNCTIONAL DISABILITY

If the mother has died, skip to Section F.

1. **Earliest time** at which mother was able to complete the listed tasks after delivery. (Please check **most applicable** box.)

	NA*	Within the following period of time after birth						Not yet able
		**3d**	**7d**	**14d**	**2wk**	**4wk**	**6wk**	
a. Baby care	1|__|	2|__|	3|__|	4|__|	5|__|	6|__|	7|__|	8|__|
b. Walk about home	1|__|	2|__|	3|__|	4|__|	5|__|	6|__|	7|__|	8|__|
Ask following questions **only** if support not available or must work								
c. Wash baby's clothes	1|__|	2|__|	3|__|	4|__|	5|__|	6|__|	7|__|	8|__|
d. Prepare meals	1|__|	2|__|	3|__|	4|__|	5|__|	6|__|	7|__|	8|__|
e. Clean house	1|__|	2|__|	3|__|	4|__|	5|__|	6|__|	7|__|	8|__|
f. Get water	1|__|	2|__|	3|__|	4|__|	5|__|	6|__|	7|__|	8|__|
g. Work in fields	1|__|	2|__|	3|__|	4|__|	5|__|	6|__|	7|__|	8|__|
h. Go to market	1|__|	2|__|	3|__|	4|__|	5|__|	6|__|	7|__|	8|__|
i. Return to	1|__|	2|__|	3|__|	4|__|	5|__|	6|__|	7|__|	8|__|

University of British Columbia<br>KLE University's JNMC & SNMC	Maternal Newborn Health Registry<br>**SUPPLEMENTAL - FOLLOW-UP FORM**	**MN03 (Supplemental)**
**Page** 5 of 5	**SUBJECT ID: |___|___|___|___|___|___|**	**Version 6 2013/07/26**

This form covers events that may have occurred since completion of the MN02 Perinatal Form (and MN02 Supplemental Perinatal Form).
If the mother was not alive at the time of MN02, complete only Sections F and G.

work or paid employment								

* *NA may include because baby has died.*

F. NEWBORN OUTCOMES for live births since completion of MN02

1. Multiple birth 1|__| Yes 2|__| No 3|__| DK

(If yes, complete Sections F and G only for twin using ID ending in 2, for triplets 3.)

2. Newborn alive when MN02 completed
1|__| Yes 2|__| No **(If no, skip to Section G)**

3. Newborn still in hospital when MN02 completed
1|__| Yes 2|__| No 3|__| Not applicable **(If no, skip to 5)**

4. Date of newborn discharge/death |__|__|-|__|__|-|__|__|__|__|
d d m m y y y y

If DK/not yet discharged, Number of days stayed in facility |__|__| d

5. Newborn had any health problems since completion of MN02
1|__| Yes 2|__| No → **Skip to Section G.**

6. Newborn health problems after MN02. (Please indicate yes/no/DK for **each**.)

	Yes	No	DK
a. Breathing problems	1|__|	2|__|	3|__|
b. Feeding problems	1|__|	2|__|	3|__|
c. Lethargy	1|__|	2|__|	3|__|
d. Coma	1|__|	2|__|	3|__|
e. Seizure (fits)	1|__|	2|__|	3|__|
f. Fever (>38°C)	1|__|	2|__|	3|__|
g. Umbilical stump infection	1|__|	2|__|	3|__|
h. Skin infection	1|__|	2|__|	3|__|
i. Jaundice	1|__|	2|__|	3|__|
j. Diarrhea/vomiting	1|__|	2|__|	3|__|
k. Bleeding	1|__|	2|__|	3|__|

7. Newborn admitted to facility for new health problems after MN02
1|__| Yes 2|__| No 3|__| DK **(If no/DK, skip to Section G)**

8. Facility name ________________________ ID |__|__|__|__|

9. Date of admission |__|__|-|__|__| -|__|__|__|__|
d d m m y y y y

10. Newborn discharged alive 1|__| Yes
2|__| No → ***Complete CLIP P VA/SA after completing this form***
3|__| NA/currently admitted **(skip to Section G)**

11. Date of newborn discharge/death |__|__|-|__|__| -|__|__|__|__|
d d m m y y y y

If DK, Number of days stayed in facility |__|__| d

G. FORM COMPLETION

1. Date visit completed: |__|__|-|__|__|-|__|__|__|__|
d d m m y y y y

2. Name of person completing form: ____________________________
a. ID: |__|__|__|__|
b. *If applicable*, Code of BA reporting birth: |__|__|__|__|

3. Location of data collection
1|__|Home 2|__|Health Center 3|__|Hospital
